# Global, regional, and national burden of meningitis, 1990–2016: a systematic analysis for the Global Burden of Disease Study 2016

**DOI:** 10.1016/S1474-4422(18)30387-9

**Published:** 2018-12

**Authors:** GBD 2016 Meningitis Collaborators, Joseph Raymond Zunt, Nicholas J Kassebaum, Natacha Blake, Linda Glennie, Claire Wright, Emma Nichols, Foad Abd-Allah, Jemal Abdela, Ahmed Abdelalim, Abdu A Adamu, Mina G. Adib, Alireza Ahmadi, Muktar Beshir Ahmed, Amani Nidhal Aichour, Ibtihel Aichour, Miloud Taki Eddine Aichour, Nadia Akseer, Rajaa M Al-Raddadi, Fares Alahdab, Kefyalew Addis Alene, Syed Mohamed Aljunid, Mohammad A AlMazroa, Khalid Altirkawi, Nelson Alvis-Guzman, Megbaru Debalkie Animut, Mina Anjomshoa, Mustafa Geleto Ansha, Rana Jawad Asghar, Euripide F G A Avokpaho, Ashish Awasthi, Hamid Badali, Aleksandra Barac, Till Winfried Bärnighausen, Quique Bassat, Neeraj Bedi, Abate Bekele Belachew, Krittika Bhattacharyya, Zulfiqar A Bhutta, Ali Bijani, Zahid A Butt, Félix Carvalho, Carlos A Castañeda-Orjuela, Abdulaal Chitheer, Jee-Young J Choi, Devasahayam J Christopher, Anh Kim Dang, Ahmad Daryani, Gebre Teklemariam Demoz, Shirin Djalalinia, Huyen Phuc Do, Manisha Dubey, Eleonora Dubljanin, Eyasu Ejeta Duken, Maysaa El Sayed Zaki, Iqbal RF Elyazar, Hamed Fakhim, Eduarda Fernandes, Florian Fischer, Takeshi Fukumoto, Morsaleh Ganji, Abadi Kahsu Gebre, Afewerki Gebremeskel, Bradford D Gessner, Sameer Vali Gopalani, Yuming Guo, Rahul Gupta, Gessessew Bugssa Hailu, Arvin Haj-Mirzaian, Samer Hamidi, Simon I. Hay, Andualem Henok, Seyed Sina Naghibi Irvani, Ravi Prakash Jha, Mikk JÜRISSON, Amaha Kahsay, Manoochehr Karami, André Karch, Amir Kasaeian, Getachew Mullu Kassa, Tesfaye Dessale Dessale Kassa, Adane Teshome Kefale, Yousef Saleh Khader, Ibrahim A. Khalil, Ejaz Ahmad Khan, Young-Ho Khang, Jagdish Khubchandani, Ruth W Kimokoti, Adnan Kisa, Faris Hasan Lami, Miriam Levi, Shanshan Li, Clement T. Loy, Marek Majdan, Azeem Majeed, Lorenzo Giovanni Mantovani, Francisco Rogerlândio Martins-Melo, Colm Mcalinden, Varshil Mehta, Addisu Melese, Ziad A Memish, Desalegn Tadese Mengistu, Getnet Mengistu, Tomislav Mestrovic, Haftay Berhane Mezgebe, Bartosz Miazgowski, Branko Milosevic, Ali H. Mokdad, Lorenzo Monasta, Ghobad Moradi, Paula Moraga, Seyyed Meysam Mousavi, Ulrich Otto Mueller, Srinivas Murthy, Ghulam Mustafa, Aliya Naheed, Gurudatta Naik, Charles Richard James Newton, Yirga Legesse Nirayo, Molly R Nixon, Richard Ofori-Asenso, Felix Akpojene Ogbo, Andrew T Olagunju, Tinuke O Olagunju, Bolajoko Olubukunola Olusanya, Justin R Ortiz, Mayowa Ojo Owolabi, Shanti Patel, Gabriel D. Pinilla-Monsalve, Maarten J Postma, Mostafa Qorbani, Alireza Rafiei, Vafa Rahimi-Movaghar, Robert C Reiner, Andre M.N. Renzaho, Mohammad Sadegh Rezai, Kedir Teji Roba, Luca Ronfani, Gholamreza Roshandel, Ali Rostami, Hosein Safari, Saeed Safari, Saeid Safiri, Rajesh Sagar, Abdallah M. Samy, Milena M Santric Milicevic, Benn Sartorius, Shahabeddin Sarvi, Monika Sawhney, Sonia Saxena, Azadeh Shafieesabet, Masood Ali Shaikh, Mehdi Sharif, Mika Shigematsu, Si Si, Eirini Skiadaresi, Mari Smith, Ranjani Somayaji, Mu'awiyyah Babale Sufiyan, Nega Yimer Tawye, Mohamad-Hani Temsah, Miguel Tortajada-Girbés, Bach Xuan Tran, Khanh Bao Tran, Kingsley Nnanna Ukwaja, Irfan Ullah, Isidora S Vujcic, Fasil Shiferaw Wagnew, Yasir Waheed, Kidu Gidey Weldegwergs, Andrea Sylvia Winkler, Charles Shey Wiysonge, Alison B. Wiyeh, Grant M.A. Wyper, Ebrahim M. Yimer, Naohiro Yonemoto, Zoubida Zaidi, Zerihun Menlkalew Zenebe, Valery L. Feigin, Theo Vos, Christopher J L Murray

## Abstract

**Background:**

Acute meningitis has a high case-fatality rate and survivors can have severe lifelong disability. We aimed to provide a comprehensive assessment of the levels and trends of global meningitis burden that could help to guide introduction, continuation, and ongoing development of vaccines and treatment programmes.

**Methods:**

The Global Burden of Diseases, Injuries, and Risk Factors (GBD) 2016 study estimated meningitis burden due to one of four types of cause: pneumococcal, meningococcal, *Haemophilus influenzae* type b, and a residual category of other causes. Cause-specific mortality estimates were generated via cause of death ensemble modelling of vital registration and verbal autopsy data that were subject to standardised data processing algorithms. Deaths were multiplied by the GBD standard life expectancy at age of death to estimate years of life lost, the mortality component of disability-adjusted life-years (DALYs). A systematic analysis of relevant publications and hospital and claims data was used to estimate meningitis incidence via a Bayesian meta-regression tool. Meningitis deaths and cases were split between causes with meta-regressions of aetiological proportions of mortality and incidence, respectively. Probabilities of long-term impairment by cause of meningitis were applied to survivors and used to estimate years of life lived with disability (YLDs). We assessed the relationship between burden metrics and Socio-demographic Index (SDI), a composite measure of development based on fertility, income, and education.

**Findings:**

Global meningitis deaths decreased by 21·0% from 1990 to 2016, from 403 012 (95% uncertainty interval [UI] 319 426–458 514) to 318 400 (265 218–408 705). Incident cases globally increased from 2·50 million (95% UI 2·19–2·91) in 1990 to 2·82 million (2·46–3·31) in 2016. Meningitis mortality and incidence were closely related to SDI. The highest mortality rates and incidence rates were found in the peri-Sahelian countries that comprise the African meningitis belt, with six of the ten countries with the largest number of cases and deaths being located within this region. *Haemophilus influenzae* type b was the most common cause of incident meningitis in 1990, at 780 070 cases (95% UI 613 585–978 219) globally, but decreased the most (–49·1%) to become the least common cause in 2016, with 397 297 cases (291 076–533 662). Meningococcus was the leading cause of meningitis mortality in 1990 (192 833 deaths [95% UI 153 358–221 503] globally), whereas other meningitis was the leading cause for both deaths (136 423 [112 682–178 022]) and incident cases (1·25 million [1·06–1·49]) in 2016. Pneumococcus caused the largest number of YLDs (634 458 [444 787–839 749]) in 2016, owing to its more severe long-term effects on survivors. Globally in 2016, 1·48 million (1·04—1·96) YLDs were due to meningitis compared with 21·87 million (18·20—28·28) DALYs, indicating that the contribution of mortality to meningitis burden is far greater than the contribution of disabling outcomes.

**Interpretation:**

Meningitis burden remains high and progress lags substantially behind that of other vaccine-preventable diseases. Particular attention should be given to developing vaccines with broader coverage against the causes of meningitis, making these vaccines affordable in the most affected countries, improving vaccine uptake, improving access to low-cost diagnostics and therapeutics, and improving support for disabled survivors. Substantial uncertainty remains around pathogenic causes and risk factors for meningitis. Ongoing, active cause-specific surveillance of meningitis is crucial to continue and to improve monitoring of meningitis burdens and trends throughout the world.

**Funding:**

Bill & Melinda Gates Foundation.

## Introduction

Meningitis is an inflammatory condition involving the membranes (meninges) covering the brain and spinal cord. It can have infectious causes, such as bacteria, mycobacteria, viruses, fungi, or parasites, or be associated with autoimmunity, cancer, or reactions to medication. Risk factors that predispose individuals to meningitis and epidemics include malnutrition,^1^ household overcrowding,^2^ HIV infection,^3^ absence of immunisation, indoor air pollution,^4^ and sickle cell disease.^5^ Most deaths due to meningitis have infectious causes, but the clinical severity of disease varies with the causative organism.^6^ Bacterial meningitis can rapidly become fatal and lead to severe disability in those who survive. Survivors might have complications, such as cognitive impairment, behavioural problems, hearing loss, motor weakness, paralysis, incoordination, or seizure disorder; although few data are available from low-resource settings,^7^ one study found as many as a quarter of survivors of bacterial meningitis had neuropsychological sequelae 3–60 months after hospital discharge.^7^, ^8^ Both bacterial and viral meningitides can place a substantial burden upon families, communities, and societies.^9^, ^10^, ^11^ Although bacterial meningitis has often been associated with persistent intellectual impairment, some viral pathogens, such as parechovirus, have also been associated with impaired developmental attainment.^12^

**Research in context**

**Evidence before this study**

Meningitis is a disease with a high case-fatality rate that is known to occur in epidemics, requires timely and appropriate diagnosis and treatment to avoid death, and can lead to lifelong disability among survivors. In addition to previous Global Burden of Diseases, Injuries and Risk Factors (GBD) studies, some studies have investigated the causes and outcomes of meningitis in large cities and countries, but not comprehensive reporting on meningitis burden by cause, age, geography, or over time across continents. We searched PubMed without language restrictions for five components of epidemiology information related to meningitis: (1) incidence of all meningitis (from database inception to December, 2013), (2) the proportion of meningitis deaths (from database inception to November, 2016) and cases (from database inception to December, 2013) due to each of its four causes (as classified in GBD), (3) case-fatality ratio and long-term mortality experience of survivors with chronic complications of meningitis (from database inception to December, 2013), (4) the proportion of survivors with long-term complications, along with the nature and distribution of those complications (from database inception to December, 2013), and (5) the distribution of sequela severity by type of long-term outcome (from database inception to December, 2013). The following search string was used in PubMed: (((“Meningitis”[MeSH] OR “Meningitis, pneumococcal”[MeSH] OR “Meningitis, Haemophilus”[MeSH] OR “Meningitis, Meningococcal”[MeSH] OR “Meningitis, viral”[MeSH] OR “Meningitis”[Title/Abstract]) AND ((“etiology”[Title/Abstract] OR “causes” Title/Abstract] OR “cause pattern”[Title/Abstract] OR “aetiology”[Title/Abstract] OR “cause”[Title/Abstract]) AND (“fatality”[Title/Abstract] OR “mortality”[Title/Abstract] OR “death”[Title/Abstract]) AND 1985/01/01[PDAT]:3000/12/31[PDAT]) AND “humans”[MeSH]).

**Added value of this study**

This study reports on incidence, mortality, and disability of overall meningitis and its subtypes for each of 195 countries, 23 age groups, and both sexes from 1990 to 2016. It leverages the strength of the overall GBD 2016 study to generate estimates of disease burden that are internally consistent within each cause and across all causes, contextualises them within the sociodemographic development spectrum, and reveals the populations with the greatest need for improved meningitis prevention and treatment. Global burden from meningitis has reduced, but substantial disparities across geographic regions and age groups persist. Cause-specific estimates allowed for examination of trends in specific infections and their relationship to vaccine programmes. By quantifying long-term disability, this study also highlights the importance of health systems and societies being prepared to support survivors of meningitis.

**Implications of all the available evidence**

This study estimates that the largest concentration of meningitis mortality remains in the meningitis belt, which includes 26 countries across sub-Saharan Africa—from Senegal in the west to Ethiopia in the east—but many other countries also continue to have a high burden of meningitis. Vaccine coverage should be increased and existing vaccination schedules optimised to maximise population protection. Incorporating meningitis vaccines into routine vaccination schedules and ensuring these vaccines are available and administered in more countries should also be considered. Development of new vaccines and reinvestment in existing vaccines will be important to address the potential of serotype replacement in pneumococcal meningitis, to deal with epidemics, and to combat forms of meningitis that are not currently preventable. In particular, use of pneumococcal conjugate or polysaccharide vaccine in more adults and use of available or broader valency meningococcal vaccines could result in marked reductions in the burden of meningitis. Enhanced availability of appropriate antibiotics and health-care services, and low-cost diagnostics at the point of care or point of surveillance, are paramount for detecting meningitis, improving survival, and reducing disability—especially in low-resource settings where the burden of meningitis is greatest. Increased ability to detect antibiotic resistance will also become more relevant as antibiotic use and resistance continue to rise. More robust vaccine coverage and cause-specific surveillance data are crucial to improve the geographical and temporal resolution of meningitis estimates going forward.

Immunisation programmes targeting the major bacterial pathogens—*Haemophilus influenzae* type b, *Neisseria meningitidis,* and *Streptococcus pneumoniae—*have successfully reduced cases of meningitis in Africa, the Americas, Asia, Australasia, and Europe.^10^, ^13^, ^14^ The *H influenzae* type b vaccine is now part of immunisation programmes across 19 countries and the pneumococcal vaccine is part of immunisation programmes across 134 countries.^15^ The MenAfriVac mass vaccination campaign delivered over 270 million doses of menin-gococcal A protein-polysaccharide conjugate (MenA) vaccine to children and young adults in 21 of the 26 countries of the meningitis belt between 2010 and 2018, which resulted in a marked decline in cases of disease;^14^ the meningitis belt was first defined as a “region south of the Sahara between latitudes 4 and 16^0^N” and was later expanded to include the Sahelian parts of Benin, Cameroon, Ethiopia, The Gambia, Ghana, Mali, and Senegal (appendix).^16^ However, maintaining control of MenA epidemics in the long term depends on these countries implementing the vaccine routinely—a strategy taken up by only seven countries within the meningitis belt to date.^14^, ^17^

Contemporary information on country-specific incidence, mortality, and long-term disease burden of meningitis by cause is needed by governments and funders to identify areas of improvement and stagnation, strengthen screening and treatment programmes, and adequately plan to support the ongoing needs of survivors. Such data can help determine country-specific immunisation needs, and countries eligible for funding can use it to prioritise needs and assist with applications for support from Gavi, the Vaccine Alliance, UNICEF, and the Bill & Melinda Gates Foundation, among others.

The Global Burden of Diseases, Injuries, and Risk Factors (GBD) 2016 study, generated comprehensive estimates of meningitis burden by cause at the national, regional, and global level from 1990 to 2016. This report details the approach, methods, and results of the GBD 2016 analysis of the global meningitis burden to highlight levels and trends by age and geography, over time, and with respect to sociodemographic development status. The aim is to increase awareness and understanding of these estimates for stakeholders who play various roles in the identification, management, and prevention of meningitis, from researchers and clinicians to policy makers, advocates, and planners of neurological services. This report also aims to highlight some limitations and gaps in the data, with a goal of catalysing collaboration to improve future global estimates for meningitis through improved data collection, timeliness, and implementation of evidence-based policies to reduce the burden of meningitis globally.

## Methods

### Overview

The GBD 2016 estimation strategy for both mortality (deaths and years of life lost [YLLs]) and non-fatal health outcomes (prevalence, incidence, and years of life lived with disability [YLDs]), began with estimation of the epidemiology of total acute meningitis and then apportioning of that total to four sub-causes. Three specific pathogens were considered separately given their relevance to public health policy, availability of effective vaccines, and comparatively robust data sources: *S pneumonia* (pneumococcal), *N meningitides* (meningococcal), and *H influenzae* type b. The remaining pathogens (eg, bacteria, fungi, and viruses) were grouped together as other meningitis; future analyses will consider specific pathogens for disaggregation from this category. Complete descriptions of GBD 2016 methods and inputs are available in corresponding summary publications.^18^, ^19^ A detailed description of methods, including an analytic flowchart, data source search and extraction criteria, and model development details with respect to meningitis, is available in the appendix.

### Demographics, definitions, and Socio-demographic Index

The overall goal of GBD estimation methods is to combine all available data into an internally consistent analytic framework for describing death and disability due to each cause of disease or injury. This methodological approach was applied to each of the 195 countries and territories (11 of which were analysed at the first subnational administrative level [eg, states in the USA and provinces in China]), 23 age groups, both sexes, and for each year from 1990 to 2016 (inclusive). In addition to deriving specific estimates for each age group, location, sex, and year, GBD 2016 updated the method for describing epidemiological transition that is based on the Socio-demographic Index (SDI), a composite metric based on total fertility rate, education, and per-capita income, that is designed to be a summary measure of societal development and is scaled between 0 and 100. All GBD locations were grouped into SDI quintiles.

### Cause-specific mortality

We estimated mortality using vital registration and verbal autopsy data that were extracted from all sources available at the time of the study and we processed the data using a set of standardised algorithms that were used across GBD 2016 to account for incompleteness, misclassification, and stochastic variability (appendix). International Classification of Diseases, ninth Revision (ICD-9) and tenth Revision (ICD-10) codes used as the case definition for meningitis were 036–036·9/A39–A39·9 (menin-gococcal meningitis), 320·0/G00·0 (*H influenzae* type b meningitis), 320·1/G00·1 (pneumococcal meningitis), and 047–049/320·2–322·9 (other meningitis). We then modelled all available data using cause of death ensemble modelling (CODEm) with ten candidate covariates: (1) the proportion of population living in meningitis belt, (2) proportion of children younger than 5 years who are underweight (≤2 weight-for-age Z scores), (3) proportion of households with improved water, (4) the Healthcare Access and Quality Index,^20^ (5) health system access, a composite of vaccine coverage and pregnancy services, (6) diphtheria-tetanus-pertussis vaccine coverage, (7) lag distributed income (in international dollars per capita), (8) MenAfriVac vaccine coverage from 2010 to 2012, (9) improved sanitation (proportion with facilities that hygienically separate human excretia from contact with humans), and (10) maternal education (years per capita). We chose the CODEm models with the highest out-of-sample predictive validity and then combined them with estimates from all other specific causes of death to ensure the sum matched total all-cause mortality for each age group, sex, location, and year. We then assigned meningitis cause mortality in a mutually exclusive and collectively exhaustive fashion using a separate set of models of the proportion of meningitis deaths due to each cause. These models were informed by a combination of vital registration and scientific literature data identified via systematic review. YLLs were calculated by multiplying age-specific deaths and remaining global life expectancy at age of death, which was estimated by GBD 2016 to be 86·6 years at birth.^18^

### Non-fatal health outcomes

Non-fatal health outcomes were informed by a series of complementary data sources and models. First, we generated internally consistent estimates of overall meningitis incidence, prevalence, remission, and mortality using DisMod-MR 2.1, a Bayesian meta-regression tool developed for GBD. This model was informed by published literature identified via systematic review and supplemented with inpatient hospital and claims data that were corrected for readmission rates and ICD code position (primary *vs* non-primary) at discharge. ICD-coded hospital data served as the reference category. We then apportioned overall prevalence and incidence to underlying causes using a second set of DisMod-MR 2.1 proportion models informed by scientific literature and surveillance data reporting on specific infectious causes. We derived viral meningitis estimates by multiplying total meningitis and the ratio of viral-to-total meningitis from inpatient hospital sources. We calculated the incidence of each long-term cause-impairment combination by age group, sex, location, and year by multiplying the cause-specific case-fatality ratio by cause-specific incidence estimates, then applying post-discharge proportions of health consequences by cause, both of which were derived from a meta-analysis.^21^ We paired these data with corresponding long-term mortality data to derive prevalence estimates of long-term meningitis complications for each population. We then paired and multiplied final prevalence estimates with corresponding disability weights from the GBD Disability Weights Survey^22^ to calculate YLDs, which we then processed through a microsimulation framework to account for comorbidity between diseases.^19^ Long-term complications of meningitis included intellectual disa-bility, motor impairment, vision problems, hearing loss, epilepsy, and behavioural problems. The sum of YLLs and YLDs is disability-adjusted life-years (DALYs), a composite measure of overall burden.

### Risk factor attribution

We calculated attributable burden of meningitis to the risk factors low birthweight and short gestation—the only two risk factors of the 84 risk factors that met GBD 2016 causal criteria for inclusion—but only for the early and late neonatal periods as these were the only age groups for which evidence was sufficient. We quantified the population attributable fractions of low birthweight and short gestation by pairing the estimated exposure to each component with the corresponding relative risk of mortality. Details on low birthweight and short gestation estimation are in the GBD 2016 Comparative Risk Assessment paper.^23^

### Uncertainty

We captured and propagated uncertainty through all calculations by sampling 1000 values (called draws) for each prevalence, death, YLL, YLD, or DALY estimate and summing draws across age, cause, and location for all intermediate calculations. 95% uncertainty intervals (UIs) were defined by the ordinal 25th and 975th draw values. We calculated change statistics on the mean values and at the draw level to derive UIs; significant increases and decreases were defined as 975 or more of the 1000 draws having a positive or negative value, respectively.

### Role of the funding source

The funder of the study had no role in study design, data collection, data analysis, data interpretation, or the writing of the report. All authors had full access to the data in the study and final responsibility for the decision to submit for publication.

## Results

Summary results for meningitis burden are presented here. Complete results for GBD 2016 by age, sex, geography, year, and cause, including further detail not provided in this manuscript, are available in a set of interactive online visualisations called GBD Compare and are available for download from the GBD Results Tool at the Global Health Data Exchange.

3268 unique data sources were used for cause-specific mortality and 1348 unique sources for the estimation of non-fatal health outcomes from meningitis. More geographical and causal detail on dataset coverage for each component of estimation is shown in the appendix. All input sources and citations for each component are available from the GBD Input Data Sources Tool. The information on all the DisMod-MR 2.1 models, including input data and model performance, are explorable online, with corresponding information for CODEm also available.

**Figure 1:**
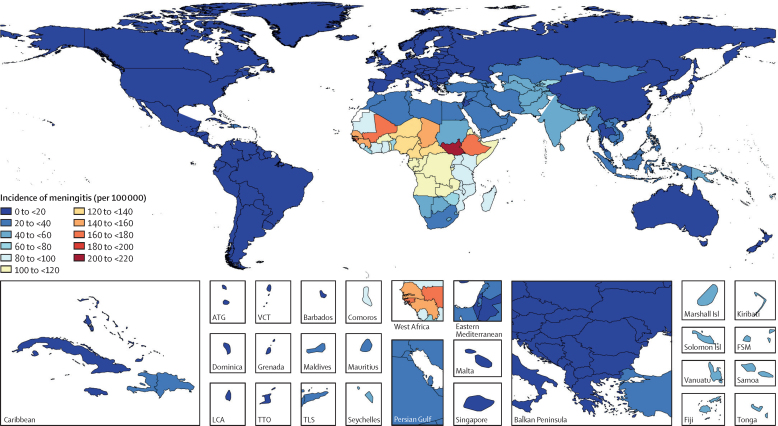
Age-standardised incidence of meningitis per 100 000 population by location for both sexes, 2016 Age-standardised incidence rate for all causes of meningitis. ATG=Antigua and Barbuda. Isl=Islands. LCA=Saint Lucia. VCT=Saint Vincent and the Grenadines. TTO=Trinidad and Tobago. TLS=Timor-Leste. FSM=Federated States of Micornesia.

Incident cases of meningitis globally increased from 2·50 million (2·19–2·91) in 1990 to 2·82 million (2·46–3·31) in 2016. Age-standardised incidence was highest in the meningitis belt—ie, the peri-Sahelian countries of sub-Saharan Africa stretching from Senegal through South Sudan and Ethiopia (figure 1). Overall incidence in 2016 varied over 350 times, from a high of 207·4 (95% UI 183·9–233·9) per 100 000 population in South Sudan to a low of 0·5 (0·4–0·7) per 100 000 population in Australia. Much of central and western sub-Saharan Africa continues to have a high burden of meningitis, with few locations showing signs of sustained improvement and several countries in particular—Gabon, Equatorial Guinea, Nigeria, South Africa, and Zambia—having much higher meningitis death rates than would be expected on the basis of their SDI.

The global number of meningitis deaths was estimated to have decreased by 21·0% from 403 012 (95% UI 319 426—458 514) in 1990 to 318 400 (265 218–408 705) in 2016 (table). Most (269 422 [84·6%] of 318 400) of these deaths occurred in countries in the low SDI (134 983 [95% UI 108 808–183 517]) and low-middle SDI (134 439 [109 866–171 733]) quintiles in 2016. The highest mortality rates were estimated for countries in the meningitis belt (table). However, of the top ten countries with the greatest absolute number of meningitis deaths—India, Nigeria, Ethiopia, Pakistan, Democratic Republic of the Congo, Uganda, Tanzania, Niger, Afghanistan, and China—four are located outside the meningitis belt (India, Pakistan, Afghanistan, China). Of the countries with at least 1000 meningitis deaths, in 2016 the countries with the largest declines were, in descending order, Brazil, China, Sudan, USA, Nepal, Haiti, Egypt, Indonesia, Myanmar, Niger, and India, all of which saw age-standardised declines in meningitis mortality of at least 50% between 1990 and 2016. Of these 11 countries, only Sudan and Niger are in the meningitis belt. Only Zimbabwe was estimated to have a significant increase in age-standardised meningitis mortality rate from 1990 to 2016 (table).

**Table:** Deaths, incidence, and DALYs for meningitis in 2016 and the percentage change of age-standardised rates by location

	**Deaths**		**Incidence**		**DALYs**	
	2016 counts	Percentage change in age-standardised rates, 1990–2016	2016 counts	Percentage change in age-standardised rates, 1990–2016	2016 counts	Percentage change in age-standardised rates, 1990–2016
**Global**	**318 400 (265 218 to 408 705)**	**−35·7 (−47·1 to −11·6)**	**2 820 772 (2 464 452 to 3 309 758)**	**−4·0 (−6·8 to −1·0)**	**21 865 891 (18 204 615 to 28 280 508)**	**−36·5 (−48·5 to −9·7)**
High SDI	4031 (3688 to 4757)	−66·8 (−70·0 to −60·6)	41 608 (36 420 to 46 278)	−28·0 (−31·0 to −25·3)	167 407 (148 625 to 205 171)	−67·4 (−70·4 to −62·9)
High-middle SDI	8814 (7599 to 11 139)	−68·9 (−75·6 to −57·0)	164 502 (141 271 to 188 401)	−26·5 (−29·7 to −23·6)	551 846 (482 463 to 670 290)	−71·2 (−77·8 to −60·3)
Middle SDI	36 002 (29 155 to 47 953)	−55·0 (−62·3 to −36·7)	381 106 (328 164 to 439 923)	−26·6 (−29·0 to −24·2)	2 248 333 (1 882 578 to 2 855 681)	−56·7 (−63·4 to −38·4)
Low–middle SDI	134 983 (108 808 to 183 517)	−42·8 (−54·2 to −21·2)	1 261 209 (1 094 765 to 1 493 485)	−15·9 (−19·1 to −12·2)	9 393 701 (7 519 284 to 12 800 909)	−44·4 (−56·8 to −19·7)
Low SDI	134 439 (109 866 to 171 733)	−35·8 (−45·8 to −19·1)	997 311 (867 721 to 1 172 673)	−14·2 (−16·5 to −12·1)	9 507 007 (7 790 912 to 12 278 609)	−43·2 (−54·1 to −21·8)
**High-income North America**	**1532 (1375 to 1845)**	**−63·0 (−66·9 to −55·4)**	**18 939 (16 776 to 20 977)**	**−26·8 (−31·6 to −21·2)**	**71 131 (63 532 to 80 226)**	**−63·4 (−66·8 to −58·6)**
Canada	107 (95 to 124)	−55·3 (−61·5 to −48·5)	2060 (1784 to 2323)	−4·7 (−8·2 to −0·8)	5279 (4568 to 5992)	−51·8 (−59·7 to −43·5)
Greenland	1 (0 to 1)	−56·9 (−68·0 to −39·5)	4 (3 to 4)	−23·1 (−26·3 to −19·6)	43 (26 to 57)	−63·4 (−73·6 to −43·9)
USA	1425 (1278 to 1731)	−63·3 (−67·2 to −55·3)	16 869 (14 990 to 18 661)	−28·6 (−33·7 to −22·5)	65 805 (58 669 to 74 353)	−64·0 (−67·6 to −59·0)
**Australasia**	**69 (63 to 84)**	**−69·4 (−73·0 to −62·5)**	**359 (317 to 417)**	**−30·9 (−34·5 to −27·2)**	**3585 (3082 to 5160)**	**−68·8 (−73·2 to −62·7)**
Australia	54 (48 to 66)	−71·4 (−75·2 to −64·4)	103 (84 to 130)	−47·4 (−53·0 to −41·8)	2738 (2299 to 4357)	−70·8 (−75·4 to −63·7)
New Zealand	15 (14 to 19)	−60·2 (−66·2 to −52·4)	257 (228 to 289)	−13·7 (−18·5 to −8·6)	847 (740 to 990)	−59·9 (−66·6 to −51·5)
**High-income Asia Pacific**	**639 (546 to 849)**	**−72·3 (−78·6 to −59·5)**	**1924 (1647 to 2198)**	**−49·5 (−52·7 to −46·4)**	**17 718 (13 601 to 35 316)**	**−75·0 (−81·7 to −62·7)**
Brunei	3 (2 to 5)	−44·2 (−60·1 to −17·5)	5 (4 to 6)	−36·4 (−40·9 to −31·4)	159 (123 to 228)	−45·1 (−60·8 to −19·3)
Japan	476 (425 to 571)	−66·3 (−69·3 to −57·9)	1525 (1309 to 1737)	−46·3 (−49·2 to −43·5)	12 339 (10 012 to 26 912)	−67·0 (−70·2 to −59·1)
Singapore	7 (5 to 12)	−85·0 (−88·9 to −75·7)	30 (25 to 35)	−50·8 (−55·2 to −45·9)	252 (187 to 456)	−84·6 (−88·2 to −75·7)
South Korea	153 (81 to 333)	−81·3 (−91·2 to −55·2)	364 (308 to 427)	−59·6 (−63·9 to −55·4)	4968 (2682 to 10 412)	−83·0 (−91·9 to −59·6)
**Western Europe**	**1630 (1462 to 1847)**	**−69·4 (−71·8 to −65·0)**	**14 495 (12 426 to 16 629)**	**−28·4 (−30·9 to −25·9)**	**61 997 (55 509 to 78 136)**	**−71·3 (−74·1 to −67·6)**
Andorra	0 (0 to 1)	−48·2 (−67·0 to −22·2)	2 (2 to 2)	−14·4 (−19·3 to −8·8)	15 (11 to 23)	−51·7 (−70·8 to −25·8)
Austria	31 (26 to 34)	−63·8 (−68·9 to −58·1)	401 (362 to 438)	−21·7 (−26·5 to −16·3)	1089 (965 to 1260)	−67·2 (−72·7 to −60·6)
Belgium	50 (43 to 56)	−51·6 (−58·8 to −43·8)	517 (450 to 583)	−5·3 (−10·9 to 0·4)	1871 (1640 to 2123)	−54·6 (−62·5 to −45·7)
Cyprus	3 (2 to 4)	−61·8 (−70·9 to −46·9)	14 (12 to 17)	−24·4 (−29·2 to −19·6)	129 (105 to 184)	−66·3 (−75·4 to −48·8)
Denmark	34 (29 to 42)	−79·2 (−83·0 to −70·7)	372 (322 to 421)	−26·6 (−31·0 to −21·7)	1053 (905 to 1336)	−80·6 (−84·1 to −74·2)
Finland	17 (15 to 21)	−74·5 (−78·2 to −66·6)	337 (295 to 379)	−10·9 (−15·0 to −6·3)	577 (497 to 734)	−73·6 (−78·0 to −67·1)
France	261 (229 to 300)	−71·2 (−75·0 to −64·7)	1788 (1543 to 2064)	−28·8 (−33·3 to −23·6)	9611 (7909 to 15 069)	−71·2 (−76·3 to −64·4)
Germany	260 (228 to 303)	−68·9 (−73·2 to −62·7)	555 (477 to 647)	−33·2 (−37·9 to −28·3)	9191 (7630 to 15 856)	−70·9 (−75·7 to −63·4)
Greece	50 (43 to 56)	−44·6 (−59·3 to −34·0)	134 (116 to 154)	−33·6 (−39·0 to −27·9)	1588 (1382 to 1923)	−49·4 (−64·5 to −37·3)
Iceland	2 (1 to 2)	−65·3 (−70·5 to −55·2)	2 (2 to 3)	−54·4 (−58·9 to −49·9)	55 (46 to 73)	−70·2 (−75·5 to −61·0)
Ireland	15 (13 to 18)	−67·0 (−72·7 to −60·6)	130 (109 to 153)	−26·9 (−32·2 to −21·7)	789 (665 to 944)	−67·5 (−73·6 to −60·2)
Israel	35 (29 to 41)	−69·2 (−74·8 to −61·6)	68 (55 to 84)	−52·1 (−56·9 to −47·6)	1434 (1196 to 1933)	−73·8 (−78·9 to −64·9)
Italy	187 (158 to 212)	−62·4 (−68·2 to −56·7)	2246 (1963 to 2527)	−8·6 (−14·3 to −3·3)	6248 (5365 to 7309)	−65·1 (−71·9 to −58·2)
Luxembourg	1 (1 to 2)	−74·8 (−78·4 to −70·2)	36 (31 to 41)	−7·6 (−13·3 to −2·2)	51 (44 to 60)	−77·4 (−81·1 to −72·8)
Malta	2 (1 to 2)	−65·0 (−70·8 to −57·4)	18 (16 to 21)	−15·3 (−19·6 to −10·5)	60 (52 to 72)	−64·1 (−70·6 to −56·4)
Netherlands	88 (76 to 98)	−72·4 (−76·6 to −67·0)	121 (103 to 144)	−47·6 (−52·4 to −42·1)	2961 (2520 to 4138)	−77·3 (−81·8 to −70·9)
Norway	19 (17 to 25)	−75·7 (−79·8 to −68·2)	214 (185 to 244)	−25·5 (−29·9 to −20·0)	707 (586 to 1008)	−79·1 (−83·2 to −72·3)
Portugal	44 (38 to 50)	−77·9 (−81·0 to −74·7)	101 (87 to 117)	−52·8 (−57·0 to −48·9)	1639 (1394 to 2434)	−81·6 (−84·8 to −77·4)
Spain	190 (167 to 215)	−73·5 (−77·2 to −69·3)	1 094 (947 to 1 246)	−33·8 (−38·3 to −28·8)	6699 (5893 to 7873)	−76·5 (−80·6 to −71·8)
Sweden	31 (27 to 38)	−65·0 (−70·0 to −56·5)	327 (254 to 396)	−34·5 (−39·3 to −29·3)	995 (852 to 1293)	−65·8 (−70·4 to −58·9)
Switzerland	25 (19 to 35)	−88·5 (−91·9 to −78·6)	340 (298 to 381)	−29·1 (−32·9 to −24·3)	721 (552 to 1125)	−87·7 (−90·9 to −79·7)
United Kingdom	287 (265 to 321)	−66·1 (−68·8 to −63·5)	5664 (4588 to 6627)	−35·9 (−39·4 to −31·8)	14 505 (13 286 to 15 800)	−68·8 (−71·2 to −66·7)
**Southern Latin America**	**642 (561 to 716)**	**−62·9 (−67·3 to −58·4)**	**2079 (1821 to 2387)**	**−38·6 (−42·3 to −34·7)**	**29 119 (25 676 to 32 338)**	**−70·0 (−74·0 to −65·7)**
Argentina	501 (435 to 558)	−57·6 (−63·5 to −52·1)	1498 (1304 to 1 732)	−38·3 (−42·4 to −33·3)	22 922 (20 151 to 25 829)	−66·5 (−71·6 to −61·0)
Chile	124 (99 to 156)	−74·0 (−79·4 to −67·0)	492 (432 to 563)	−40·1 (−44·3 to −35·7)	5615 (4610 to 6827)	−78·3 (−82·8 to −72·7)
Uruguay	18 (15 to 24)	−79·5 (−83·6 to −66·7)	89 (77 to 101)	−42·9 (−47·4 to −38·4)	581 (474 to 829)	−83·7 (−87·9 to −74·0)
**Eastern Europe**	**2084 (1680 to 614)**	**−53·0 (−62·4 to −41·2)**	**35 284 (30 821 to 39 773)**	**−18·0 (−21·9 to −14·3)**	**119 384 (98 898 to 141 302)**	**−57·0 (−64·2 to −48·4)**
Belarus	54 (44 to 74)	−69·4 (−76·6 to −58·4)	1552 (1358 to 1755)	−13·9 (−19·0 to −8·8)	3566 (2955 to 4501)	−68·5 (−75·7 to −58·9)
Estonia	7 (6 to 8)	−74·8 (−79·3 to −68·2)	205 (178 to 231)	−16·3 (−20·9 to −11·6)	372 (314 to 443)	−73·0 (−77·6 to −67·1)
Latvia	10 (9 to 15)	−80·7 (−84·9 to −71·0)	287 (255 to 318)	−27·3 (−31·6 to −23·1)	598 (486 to 762)	−78·9 (−83·6 to −72·5)
Lithuania	20 (17 to 24)	−63·2 (−68·7 to −53·7)	428 (386 to 478)	−23·4 (−28·4 to −17·9)	1040 (903 to 1 240)	−62·0 (−67·8 to −55·1)
Moldova	30 (24 to 37)	−79·8 (−85·4 to −71·7)	647 (559 to 732)	−25·7 (−30·1 to −20·5)	1955 (1561 to 2351)	−79·3 (−84·8 to −71·5)
Russia	1534 (1160 to 2019)	−51·1 (−63·7 to −35·6)	24 800 (21 641 to 27 949)	−17·7 (−22·1 to −13·2)	85 083 (67 234 to 104 629)	−57·0 (−65·9 to −47·1)
Ukraine	430 (323 to 569)	−47·7 (−63·3 to −25·8)	7366 (6411 to 8332)	−18·2 (−23·0 to −13·4)	26 771 (20 737 to 35 223)	−48·0 (−63·1 to −24·1)
**Central Europe**	**521 (471 to 618)**	**−73·5 (−76·2 to −68·9)**	**9334 (8144 to 10 411)**	**−23·2 (−26·3 to −20·3)**	**30 604 (26 870 to 35 078)**	**−72·7 (−75·9 to −68·4)**
Albania	27 (18 to 48)	−65·7 (−80·2 to −37·0)	318 (276 to 363)	−19·6 (−24·0 to −15·1)	1652 (1114 to 2678)	−68·0 (−81·9 to −38·8)
Bosnia and Herzegovina	14 (10 to 23)	−57·1 (−71·1 to −27·4)	355 (307 to 403)	−6·5 (−10·7 to −1·6)	946 (755 to 1 372)	−53·5 (−67·0 to −29·6)
Bulgaria	45 (37 to 54)	−54·5 (−64·3 to −41·7)	705 (614 to 791)	−11·2 (−15·8 to −6·8)	2558 (2088 to 3112)	−54·0 (−64·5 to −39·2)
Croatia	17 (14 to 21)	−73·2 (−78·0 to −66·3)	404 (366 to 442)	−16·0 (−20·8 to −10·2)	918 (770 to 1 081)	−68·8 (−74·2 to −62·7)
Czech Republic	41 (36 to 50)	−70·5 (−75·6 to −65·7)	965 (848 to 1 073)	−20·4 (−25·3 to −16·1)	2292 (1936 to 2668)	−65·9 (−71·9 to −60·6)
Hungary	62 (53 to 76)	−69·6 (−75·2 to −62·2)	932 (805 to 1 051)	−21·3 (−26·7 to −16·5)	2863 (2441 to 3409)	−67·8 (−74·0 to −60·4)
Macedonia	9 (7 to 13)	−70·1 (−81·0 to −47·8)	205 (177 to 232)	−14·2 (−19·2 to −8·9)	708 (544 to 957)	−69·6 (−80·4 to −47·6)
Montenegro	2 (1 to 3)	−49·7 (−67·1 to −23·4)	61 (53 to 70)	−3·5 (−8·1 to 1·6)	130 (104 to 168)	−48·1 (−65·2 to −23·7)
Poland	158 (133 to 189)	−75·7 (−79·8 to −69·9)	2274 (1 978 to 2 578)	−33·6 (−39·6 to −27·2)	8962 (7447 to 10 700)	−74·9 (−79·5 to −69·3)
Romania	78 (67 to 104)	−82·7 (−85·6 to −77·8)	1417 (1220 to 1598)	−28·9 (−32·9 to −24·2)	5176 (4405 to 6103)	−82·3 (−85·3 to −78·2)
Serbia	35 (27 to 47)	−56·3 (−68·2 to −36·3)	731 (638 to 822)	−17·4 (−22·7 to −12·3)	2312 (1956 to 2822)	−56·5 (−67·9 to −37·5)
Slovakia	27 (19 to 35)	−41·6 (−62·0 to −19·7)	697 (615 to 781)	−10·6 (−15·7 to −5·9)	1716 (1340 to 2156)	−41·2 (−60·2 to −18·8)
Slovenia	7 (6 to 8)	−74·0 (−78·1 to −68·2)	271 (239 to 302)	−8·0 (−13·1 to −3·3)	371 (308 to 445)	−66·2 (−71·3 to −60·4)
**Central Asia**	**1385 (1159 to 1774)**	**−61·9 (−68·6 to −51·5)**	**39 069 (33 907 to 44 828)**	**−7·8 (−11·2 to −4·7)**	**111 906 (93 309 to 139 943)**	**−60·6 (−67·4 to −49·9)**
Armenia	11 (9 to 13)	−62·3 (−68·2 to −55·4)	1001 (857 to 1143)	8·1 (3·0 to 13·0)	1198 (971 to 1438)	−47·2 (−54·5 to −39·7)
Azerbaijan	128 (89 to 194)	−31·5 (−56·1 to 0·7)	3803 (3286 to 4367)	2·5 (−2·7 to 8·2)	10 347 (7725 to 15 193)	−32·8 (−54·7 to −2·8)
Georgia	35 (29 to 43)	−54·8 (−64·5 to −42·6)	768 (664 to 882)	−17·3 (−21·5 to −12·6)	2591 (2164 to 3119)	−51·5 (−60·6 to −39·8)
Kazakhstan	198 (158 to 240)	−58·0 (−67·2 to −47·2)	7035 (6063 to 8172)	−6·0 (−11·2 to −0·3)	15 581 (12 468 to 19 048)	−57·9 (−66·8 to −47·6)
Kyrgyzstan	71 (59 to 88)	−77·0 (−81·6 to −69·9)	4819 (4133 to 5595)	−3·7 (−9·3 to 1·6)	6861 (5692 to 8261)	−72·4 (−77·8 to −65·5)
Mongolia	52 (28 to 109)	−85·7 (−94·4 to −45·5)	1104 (937 to 1296)	−43·7 (−48·4 to −39·4)	3813 (2265 to 6870)	−86·9 (−94·5 to −54·8)
Tajikistan	459 (305 to 750)	−46·0 (−61·9 to −17·7)	4656 (4069 to 5402)	−14·2 (−18·7 to −9·1)	34 706 (22 943 to 56 596)	−48·4 (−64·3 to −17·7)
Turkmenistan	95 (72 to 126)	−69·4 (−77·2 to −59·1)	2359 (2039 to 2753)	−16·3 (−21·1 to −11·3)	8101 (6257 to 10 643)	−67·0 (−75·7 to −55·0)
Uzbekistan	336 (264 to 449)	−73·4 (−79·4 to −62·0)	13 524 (11 680 to 15 690)	−12·2 (−17·2 to −7·3)	28 707 (22 863 to 36 717)	−69·9 (−76·3 to −60·0)
**Central Latin America**	**2324 (2137 to 2679)**	**−64·3 (−67·1 to −60·6)**	**24 075 (20 186 to 28 016)**	**−35·8 (−38·7 to −33·1)**	**148 319 (133 549 to 171 064)**	**−67·8 (−70·7 to −63·5)**
Colombia	457 (400 to 546)	−71·1 (−75·9 to −66·3)	5804 (5068 to 6560)	−22·8 (−27·0 to −18·3)	27 473 (23 383 to 33 868)	−74·1 (−78·6 to −68·4)
Costa Rica	37 (32 to 45)	−53·6 (−60·7 to −43·9)	586 (506 to 660)	−7·0 (−11·4 to −3·0)	2058 (1707 to 2556)	−52·6 (−62·1 to −40·2)
El Salvador	105 (82 to 156)	−57·0 (−66·9 to −36·9)	762 (665 to 860)	−14·8 (−19·1 to −9·8)	5483 (4139 to 8511)	−64·7 (−74·3 to −42·5)
Guatemala	323 (267 to 383)	−27·9 (−48·2 to −8·4)	2917 (2541 to 3321)	3·4 (−1·7 to 8·3)	21 114 (17 859 to 25 035)	−36·2 (−50·4 to −21·1)
Honduras	183 (122 to 306)	−42·6 (−59·8 to −16·2)	1162 (1004 to 1318)	1·5 (−2·5 to 5·9)	11 049 (6963 to 20 345)	−49·2 (−65·5 to −16·9)
Mexico	749 (700 to 861)	−71·3 (−73·4 to −67·1)	7598 (5632 to 9609)	−58·2 (−63·0 to −53·7)	48 608 (44 622 to 55 305)	−74·8 (−76·9 to −71·3)
Nicaragua	83 (59 to 136)	−66·3 (−76·9 to −38·0)	759 (657 to 872)	−24·4 (−28·7 to −19·9)	5833 (4106 to 9803)	−71·3 (−80·9 to −41·7)
Panama	51 (42 to 66)	−58·5 (−66·5 to −46·5)	524 (454 to 592)	−10·7 (−14·5 to −6·6)	3274 (2574 to 4320)	−58·2 (−67·7 to −44·4)
Venezuela	335 (280 to 426)	−59·7 (−67·3 to −47·0)	3962 (3403 to 4521)	−15·5 (−20·1 to −11·2)	23 426 (19 686 to 29 027)	−59·0 (−66·5 to −48·0)
**Andean Latin America**	**716 (557 to 932)**	**−60·9 (−69·9 to −42·4)**	**4861 (4150 to 5769)**	**−24·3 (−28·0 to −20·2)**	**49 843 (38 853 to 65 795)**	**−64·5 (−73·5 to −44·9)**
Bolivia	227 (164 to 324)	−64·3 (−75·9 to −42·0)	1088 (922 to 1295)	−28·4 (−32·7 to −24·2)	16 299 (11 403 to 23 661)	−68·0 (−79·8 to −43·8)
Ecuador	144 (126 to 168)	−65·4 (−70·2 to −59·9)	1181 (1003 to 1381)	−30·2 (−35·4 to −25·1)	9631 (8273 to 11 374)	−67·6 (−72·9 to −61·0)
Peru	345 (211 to 515)	−55·8 (−74·0 to −23·8)	2592 (2197 to 3086)	−19·5 (−24·5 to −14·7)	23 912 (14 841 to 36 285)	−60·4 (−77·5 to −27·1)
**Caribbean**	**1610 (1116 to 2556)**	**−57·6 (−73·4 to −32·4)**	**8213 (7118 to 9497)**	**−15·3 (−18·7 to −12·3)**	**118 801 (77 334 to 198 723)**	**−57·0 (−74·2 to −26·3)**
Antigua and Barbuda	1 (1 to 1)	−52·1 (−61·1 to −36·9)	12 (11 to 14)	−15·8 (−20·6 to −10·9)	57 (46 to 72)	−46·8 (−58·2 to −31·9)
The Bahamas	5 (4 to 6)	−59·2 (−70·8 to −40·1)	55 (48 to 64)	−11·8 (−16·8 to −6·7)	247 (193 to 335)	−61·3 (−74·2 to −39·8)
Barbados	4 (4 to 6)	−53·4 (−63·6 to −35·7)	38 (33 to 43)	−19·0 (−23·5 to −14·0)	168 (136 to 213)	−51·2 (−65·0 to −29·2)
Belize	4 (3 to 6)	−52·7 (−63·6 to −36·3)	61 (52 to 72)	−9·6 (−14·5 to −3·8)	259 (186 to 373)	−62·8 (−72·9 to −47·3)
Bermuda	0 (0 to 0)	−77·5 (−82·6 to −68·4)	9 (8 to 11)	−6·2 (−11·7 to −0·4)	15 (13 to 19)	−73·3 (−78·9 to −64·9)
Cuba	98 (85 to 135)	−84·1 (−87·7 to −77·2)	759 (661 to 864)	−39·5 (−44·1 to −35·1)	4485 (3918 to 5459)	−85·1 (−88·6 to −79·9)
Dominica	1 (1 to 1)	−44·4 (−55·0 to −28·9)	11 (9 to 12)	−4·5 (−9·4 to 0·9)	53 (42 to 66)	−37·7 (−53·0 to −17·1)
Dominican Republic	244 (183 to 351)	−59·9 (−72·7 to −36·1)	2855 (2486 to 3299)	−13·5 (−18·1 to −8·7)	16 669 (12 119 to 23 666)	−62·7 (−75·8 to −36·9)
Grenada	1 (1 to 2)	−54·1 (−65·1 to −29·9)	16 (13 to 18)	−6·8 (−12·8 to −1·7)	63 (51 to 84)	−55·0 (−67·4 to −31·3)
Guyana	10 (8 to 13)	−56·7 (−64·8 to −41·4)	108 (93 to 126)	−10·2 (−15·0 to −5·0)	562 (470 to 707)	−58·8 (−66·9 to −46·6)
Haiti	1139 (671 to 2043)	−61·0 (−78·0 to −26·7)	2800 (2372 to 3355)	−25·9 (−30·5 to −21·6)	90 530 (50 270 to 169 298)	−63·3 (−80·7 to −26·5)
Jamaica	50 (33 to 84)	−39·9 (−62·5 to −0·9)	447 (388 to 521)	−9·2 (−13·6 to −4·3)	2848 (1803 to 4797)	−44·5 (−67·0 to −3·0)
Puerto Rico	19 (16 to 27)	−70·3 (−76·4 to −56·2)	465 (400 to 527)	−5·8 (−10·6 to −0·6)	977 (819 to 1249)	−67·3 (−72·9 to −57·5)
Saint Lucia	2 (2 to 3)	−59·8 (−68·0 to −43·8)	23 (20 to 26)	−14·3 (−19·5 to −9·2)	102 (86 to 131)	−58·2 (−70·0 to −38·9)
Saint Vincent and the Grenadines	2 (1 to 3)	−61·2 (−70·6 to −35·3)	16 (14 to 19)	−19·5 (−23·9 to −15·0)	86 (67 to 121)	−63·2 (−73·9 to −40·6)
Suriname	13 (9 to 20)	−40·5 (−60·4 to −10·2)	83 (72 to 96)	−12·2 (−17·0 to −7·3)	723 (491 to 1 101)	−44·9 (−66·6 to −10·0)
Trinidad and Tobago	16 (12 to 24)	−63·6 (−73·2 to −40·2)	162 (140 to 185)	−15·3 (−20·5 to −9·8)	773 (607 to 1 124)	−60·4 (−71·7 to −37·5)
Virgin Islands	1 (1 to 1)	−42·7 (−59·4 to −19·9)	14 (12 to 16)	6·9 (2·0 to 13·2)	28 (22 to 38)	−41·7 (−57·2 to −22·5)
**Tropical Latin America**	**2503 (2332 to 2757)**	**−75·4 (−77·4 to −72·4)**	**13 546 (11 483 to 15 628)**	**−65·3 (−67·7 to −62·6)**	**156 925 (145 595 to 172 468)**	**−78·1 (−80·2 to −75·0)**
Brazil	2382 (2208 to 2616)	−76·1 (−78·1 to −73·2)	12 819 (10 880 to 14 778)	−66·4 (−68·7 to −63·7)	148 971 (138 379 to 163 331)	−78·7 (−80·7 to −75·9)
Paraguay	121 (89 to 181)	−41·8 (−57·3 to −17·4)	727 (622 to 853)	−17·5 (−22·3 to −12·7)	7954 (5582 to 12 473)	−48·0 (−64·3 to −22·9)
**East Asia**	**7290 (5352 to 10 245)**	**−74·6 (−79·8 to −60·2)**	**49 506 (39 811 to 59 016)**	**−66·8 (−69·5 to −64·2)**	**424 020 (329 808 to 532 986)**	**−75·9 (−80·6 to −63·0)**
China	6907 (5003 to 9712)	−75·5 (−80·5 to −61·1)	46 375 (37 051 to 55 379)	−68·3 (−70·9 to −65·6)	400 735 (304 552 to 507 387)	−76·9 (−81·4 to −63·7)
North Korea	260 (185 to 381)	−11·3 (−43·5 to 34·5)	1962 (1673 to 2312)	−4·2 (−9·3 to 1·2)	17 753 (11 887 to 25 999)	−6·4 (−42·4 to 43·6)
Taiwan (province of China)	123 (83 to 158)	−54·4 (−64·6 to −39·7)	1169 (1009 to 1335)	−16·1 (−20·7 to −10·6)	5532 (4363 to 6978)	−48·0 (−58·6 to −31·6)
**Southeast Asia**	**12886 (9541 to 15063)**	**−51·2 (−59·6 to −34·9)**	**199 475 (171 557 to 233 509)**	**−16·7 (−19·3 to −14·3)**	**947 799 (738 389 to 1 100 920)**	**−53·4 (−62·3 to −35·5)**
Cambodia	424 (260 to 636)	−64·9 (−76·6 to −48·2)	5580 (4773 to 6580)	−28·6 (−33·9 to −22·8)	31 847 (19 137 to 47 061)	−67·1 (−78·9 to −45·3)
Indonesia	4313 (2041 to 5731)	−54·5 (−66·4 to −25·9)	78 018 (66 547 to 90 966)	−26·7 (−29·3 to −23·8)	347 207 (190 560 to 456 825)	−57·5 (−68·7 to −26·9)
Laos	492 (226 to 904)	−61·6 (−79·9 to −30·1)	3539 (3044 to 4164)	−24·3 (−28·7 to −20·0)	40 578 (17 976 to 76 316)	−62·0 (−81·1 to −21·6)
Malaysia	516 (378 to 749)	−47·0 (−59·0 to −28·6)	10 400 (8623 to 12 509)	−18·9 (−25·0 to −12·1)	31 375 (24 772 to 42 105)	−48·6 (−61·7 to −27·6)
Maldives	1 (1 to 2)	−86·2 (−91·5 to −69·0)	105 (90 to 122)	−32·5 (−37·4 to −27·8)	157 (125 to 200)	−85·4 (−90·9 to −68·6)
Mauritius	10 (9 to 12)	−55·4 (−62·8 to −45·9)	310 (266 to 355)	−1·1 (−6·6 to 4·3)	764 (653 to 893)	−47·3 (−56·3 to −37·0)
Myanmar	1608 (1104 to 2345)	−54·2 (−70·0 to −22·5)	17 967 (15 638 to 20 916)	−21·3 (−26·3 to −15·8)	110 563 (77 684 to 158 797)	−57·0 (−74·1 to −19·8)
Philippines	3019 (2553 to 3520)	−48·3 (−56·1 to −39·7)	39 895 (34 447 to 46 092)	−12·8 (−17·9 to −7·0)	236 309 (201 758 to 276 233)	−48·2 (−57·0 to −39·0)
Sri Lanka	485 (316 to 720)	−50·9 (−63·4 to −29·9)	8099 (6787 to 9392)	−14·0 (−19·8 to −8·4)	20 848 (15 425 to 32 408)	−60·0 (−71·0 to −39·7)
Seychelles	4 (3 to 7)	−61·8 (−71·5 to −45·2)	38 (33 to 43)	−10·7 (−15·6 to −6·4)	213 (170 to 310)	−68·1 (−76·9 to −53·1)
Thailand	1 068 (701 to 1 383)	−47·9 (−67·6 to −26·5)	9259 (8030 to 10 518)	−11·0 (−16·9 to −5·3)	55 002 (40 198 to 67 758)	−55·2 (−68·5 to −35·7)
Timor–Leste	35 (21 to 57)	−72·3 (−84·2 to −42·3)	476 (402 to 573)	−26·3 (−31·4 to −20·7)	2878 (1696 to 4667)	−73·8 (−85·6 to −40·1)
Vietnam	909 (697 to 1288)	−59·7 (−72·3 to −34·8)	25 543 (21 869 to 29 873)	3·9 (−2·0 to 10·3)	69 865 (54 595 to 96 176)	−59·1 (−71·7 to −32·6)
**Oceania**	**667 (468 to 1010)**	**−27·0 (−46·9 to 2·5)**	**6238 (5388 to 7304)**	**−8·2 (−11·8 to −4·9)**	**50 003 (34 464 to 77 977)**	**−26·3 (−50·1 to 12·6)**
American Samoa	1 (1 to 1)	−46·5 (−61·2 to −19·1)	37 (31 to 43)	−2·3 (−7·5 to 3·2)	71 (58 to 90)	−38·9 (−53·3 to −16·1)
Federated States of Micronesia	2 (2 to 4)	−49·8 (−69·2 to −23·6)	46 (39 to 53)	−11·5 (−16·1 to −6·8)	184 (129 to 278)	−51·7 (−70·9 to −26·5)
Fiji	25 (18 to 36)	−12·4 (−46·6 to 34·8)	338 (293 to 384)	2·3 (−2·8 to 8·1)	1860 (1353 to 2568)	0·1 (−42·3 to 59·8)
Guam	2 (1 to 2)	−40·1 (−59·9 to 4·8)	77 (66 to 89)	4·2 (−0·6 to 9·7)	144 (113 to 192)	−27·4 (−49·2 to 8·2)
Kiribati	15 (10 to 23)	−23·4 (−45·1 to 11·2)	69 (59 to 81)	−22·4 (−26·8 to −18·0)	1078 (680 to 1731)	−25·5 (−50·8 to 21·0)
Marshall Islands	2 (1 to 3)	−44·6 (−65·6 to −20·3)	38 (32 to 45)	−11·1 (−15·4 to −6·2)	168 (111 to 254)	−44·4 (−64·8 to −17·6)
Northern Mariana Islands	0 (0 to 1)	−31·7 (−50·1 to −8·0)	61 (51 to 71)	6·0 (1·1 to 10·7)	62 (47 to 78)	−14·7 (−29·7 to 1·0)
Papua New Guinea	578 (387 to 925)	−30·4 (−51·4 to 2·7)	4641 (3994 to 5478)	−10·3 (−14·4 to −6·2)	43 057 (27 755 to 71 087)	−32·1 (−56·1 to 9·6)
Samoa	3 (2 to 6)	−43·7 (−62·9 to −19·4)	102 (86 to 122)	−4·6 (−9·3 to 1·2)	268 (171 to 425)	−40·6 (−61·1 to −14·4)
Solomon Islands	19 (12 to 30)	−27·9 (−52·6 to 5·2)	319 (275 to 378)	−7·8 (−11·8 to −3·4)	1474 (956 to 2262)	−22·1 (−48·9 to 14·6)
Tonga	9 (6 to 12)	−24·9 (−46·5 to 3·6)	72 (62 to 84)	−6·7 (−11·3 to −1·7)	572 (387 to 829)	−22·6 (−49·1 to 17·7)
Vanuatu	11 (7 to 17)	−18·8 (−47·2 to 16·3)	156 (134 to 183)	−3·4 (−8·1 to 1·5)	846 (528 to 1349)	−8·9 (−39·4 to 36·0)
**North Africa and Middle East**	**14 706 (11 041 to 21 129)**	**−48·7 (−63·0 to −26·3)**	**205 410 (174 410 to 244 254)**	**−15·5 (−19·4 to −12·2)**	**1 152 435 (858 927 to 1 677 016)**	**−51·5 (−66·2 to −26·1)**
Afghanistan	7302 (4522 to 12 624)	−31·0 (−55·5 to 16·7)	22 785 (19 182 to 27 754)	−22·0 (−26·4 to −17·6)	575 559 (343 317 to 1 031 288)	−36·3 (−62·4 to 25·0)
Algeria	368 (248 to 557)	−58·6 (−72·7 to −29·9)	13 062 (11 049 to 15 677)	−16·3 (−22·3 to −10·0)	27 630 (19 210 to 39 243)	−60·7 (−75·3 to −30·4)
Bahrain	6 (5 to 9)	−54·2 (−66·3 to −32·3)	316 (267 to 373)	−1·8 (−7·2 to 4·2)	565 (449 to 731)	−50·1 (−61·9 to −28·5)
Egypt	1121 (687 to 1825)	−56·1 (−71·4 to −31·1)	27 985 (22 189 to 35 040)	−10·9 (−19·5 to −1·4)	88 118 (57 888 to 144 620)	−59·1 (−72·9 to −33·3)
Iran	875 (637 to 1 354)	−53·3 (−68·0 to −25·0)	21 523 (18 344 to 25 420)	−13·5 (−20·5 to −6·3)	58 229 (41 929 to 87 211)	−62·0 (−75·9 to −33·2)
Iraq	1549 (865 to 2 485)	−38·2 (−60·6 to 0·1)	21 154 (17 881 to 26 099)	−11·5 (−16·7 to −5·8)	119 895 (66 553 to 196 769)	−45·3 (−67·4 to 2·8)
Jordan	56 (42 to 73)	−46·1 (−61·1 to −20·7)	1760 (1416 to 2199)	−0·8 (−9·9 to 7·4)	5133 (4087 to 6561)	−43·6 (−58·5 to −16·3)
Kuwait	12 (9 to 15)	−39·3 (−54·5 to −19·8)	947 (799 to 1110)	2·4 (−3·0 to 7·9)	1398 (1092 to 1707)	−31·7 (−43·4 to −18·4)
Lebanon	31 (20 to 55)	−63·7 (−77·3 to −35·1)	1326 (1125 to 1562)	2·8 (−4·8 to 10·5)	2393 (1736 to 3595)	−58·0 (−71·5 to −29·4)
Libya	43 (31 to 68)	−47·9 (−63·0 to −25·2)	1557 (1312 to 1870)	−2·3 (−9·4 to 5·4)	3241 (2423 to 4549)	−52·2 (−66·0 to −25·4)
Morocco	586 (376 to 1152)	−65·7 (−80·7 to −30·8)	9381 (8055 to 11 094)	−16·6 (−22·2 to −10·5)	46013 (29075 to 90201)	−67·7 (−82·4 to −31·3)
Oman	20 (15 to 29)	−52·7 (−69·5 to −30·2)	1246 (1044 to 1495)	−1·4 (−6·8 to 4·7)	1825 (1423 to 2414)	−59·1 (−71·3 to −32·0)
Palestine	51 (34 to 77)	−61·5 (−76·4 to −20·5)	2296 (1923 to 2809)	0·1 (−5·9 to 7·6)	4429 (3027 to 6547)	−66·6 (−82·0 to −29·5)
Qatar	6 (4 to 8)	−34·0 (−64·5 to 6·1)	484 (414 to 562)	7·2 (1·8 to 13·2)	778 (611 to 983)	−30·0 (−55·7 to 0·2)
Saudi Arabia	113 (71 to 147)	−56·3 (−67·2 to −38·9)	7517 (6249 to 8920)	13·1 (7·9 to 18·5)	9309 (7327 to 11 648)	−59·5 (−69·3 to −43·0)
Sudan	1104 (554 to 2192)	−65·6 (−78·1 to −46·0)	22 315 (19 044 to 26 916)	−5·0 (−11·6 to 2·2)	90 855 (46 038 to 175 454)	−66·4 (−79·3 to −43·8)
Syria	256 (193 to 347)	−60·5 (−72·9 to −40·5)	5201 (4379 to 6248)	−15·2 (−22·0 to −9·7)	18 910 (14 450 to 25 756)	−66·5 (−78·3 to −44·2)
Tunisia	89 (61 to 146)	−61·8 (−74·6 to −37·4)	2795 (2394 to 3312)	−8·1 (−14·3 to −1·3)	6111 (4485 to 8689)	−63·9 (−76·0 to −38·0)
Turkey	443 (337 to 610)	−91·5 (−95·8 to −68·7)	28 783 (23 890 to 34 399)	−38·7 (−44·1 to −33·2)	40 557 (32 330 to 51 230)	−91·1 (−95·4 to −68·5)
United Arab Emirates	143 (91 to 225)	−32·0 (−50·1 to −5·9)	2794 (2296 to 3362)	−9·6 (−15·4 to −4·1)	8694 (6224 to 12 764)	−39·9 (−57·2 to −16·2)
Yemen	531 (310 to 913)	−58·0 (−71·3 to −36·8)	10 004 (8345 to 12 510)	−25·3 (−31·8 to −19·0)	42 668 (24 668 to 72 747)	−63·5 (−77·3 to −35·1)
**South Asia**	**81 962 (66 471 to 116 920)**	**−49·6 (−60·9 to −24·8)**	**767 722 (673 756 to 885 020)**	**−28·3 (−30·7 to −25·5)**	**5 261 646 (4 304 995 to 7 010 042)**	**−51·9 (−62·5 to −25·6)**
Bangladesh	1396 (577 to 2254)	−3·0 (−77·0 to 141·5)	139 943 (120 236 to 161 523)	10·9 (6·6 to 16·0)	157 306 (95 836 to 221 249)	−10·5 (−64·0 to 71·3)
Bhutan	28 (14 to 42)	−70·5 (−80·6 to −50·5)	398 (345 to 464)	−28·1 (−33·1 to −22·9)	1967 (1162 to 2896)	−72·9 (−83·8 to −48·6)
India	63 001 (49 501 to 96 604)	−51·7 (−63·4 to −25·3)	504 959 (445 468 to 582 296)	−36·6 (−38·7 to −34·0)	3 795 380 (3 061 776 to 5 402 776)	−55·0 (−65·2 to −26·6)
Nepal	1201 (605 to 1876)	−62·2 (−73·4 to −41·6)	16 619 (14 360 to 19 692)	−26·2 (−31·7 to −21·1)	78 822 (45 701 to 129 989)	−67·5 (−79·5 to −39·3)
Pakistan	16 335 (11 443 to 23 598)	−39·6 (−56·4 to −10·4)	105 803 (91 301 to 125 310)	−11·1 (−15·5 to −5·5)	1 228 172 (863 627 to 1 806 081)	−44·2 (−62·6 to −6·2)
**Southern sub-Saharan Africa**	**5327 (4058 to 6810)**	**9·4 (−19·2 to 36·4)**	**35 745 (31 308 to 41 372)**	**−6·8 (−9·6 to −4·1)**	**325 045 (258 725 to 412 961)**	**−3·6 (−25·6 to 19·7)**
Botswana	88 (41 to 168)	−19·5 (−57·0 to 20·4)	1245 (1031 to 1483)	−0·9 (−6·9 to 4·9)	5132 (2679 to 9202)	−28·2 (−58·7 to 4·2)
Lesotho	150 (98 to 257)	14·0 (−22·2 to 65·5)	1243 (1081 to 1427)	13·5 (7·9 to 18·8)	9498 (6397 to 15 203)	7·2 (−24·6 to 52·3)
Namibia	116 (71 to 198)	−30·3 (−51·4 to −2·3)	1335 (1146 to 1608)	−3·1 (−8·2 to 2·2)	7566 (4759 to 12369)	−32·2 (−52·3 to 0·6)
South Africa	2625 (1709 to 3245)	−8·1 (−40·9 to 16·1)	19 011 (16 694 to 21 740)	−15·9 (−18·6 to −13·0)	149 592 (105 738 to 179 563)	−24·5 (−47·2 to −6·7)
Swaziland	85 (55 to 133)	−5·2 (−33·3 to 28·7)	876 (745 to 1053)	13·8 (7·8 to 20·0)	5 927 (3862 to 9033)	−4·5 (−34·0 to 36·4)
Zimbabwe	2262 (1613 to 3114)	50·8 (3·4 to 128·0)	12 035 (10 420 to 14 118)	1·8 (−3·5 to 7·5)	147 331 (105 080 to 207 602)	39·1 (−1·9 to 95·5)
**Western sub-Saharan Africa**	**90 003 (66 791 to 129 320)**	**−33·3 (−46·0 to −14·3)**	**650 298 (552 289 to 792 435)**	**−16·5 (−20·5 to −12·4)**	**6 635 561 (4 913 280 to 9 716 099)**	**−40·5 (−54·4 to −16·4)**
Benin	2237 (1591 to 3475)	−39·3 (−54·3 to −19·3)	10 775 (9 093 to 12 879)	−27·4 (−31·8 to −22·9)	152 272 (105 407 to 242 000)	−52·0 (−66·6 to −26·8)
Burkina Faso	5625 (3908 to 8109)	−36·4 (−51·4 to −15·3)	30 343 (26 217 to 36 191)	−18·6 (−23·8 to −14·0)	415 218 (277 744 to 616 681)	−42·1 (−59·9 to −13·5)
Cameroon	4935 (3592 to 6703)	−17·9 (−36·2 to 3·8)	39 867 (33 363 to 49 427)	−3·6 (−9·9 to 4·0)	348 226 (248 222 to 484 426)	−22·4 (−42·2 to 6·8)
Cape Verde	21 (15 to 34)	−61·8 (−76·5 to −30·6)	563 (482 to 672)	−12·4 (−17·5 to −6·6)	1270 (938 to 2007)	−67·1 (−80·1 to −40·3)
Chad	4788 (3361 to 6674)	−17·1 (−35·5 to 9·3)	33 678 (28 804 to 39 983)	−4·7 (−9·5 to 0·9)	364 952 (249 949 to 513 831)	−19·3 (−42·5 to 17·5)
Côte d'Ivoire	4154 (3058 to 5449)	−16·2 (−32·6 to 0·9)	24 954 (21 507 to 30 087)	−12·3 (−17·1 to −7·8)	277 444 (200 720 to 383 842)	−23·3 (−43·0 to 2·1)
The Gambia	364 (237 to 602)	−23·7 (−42·1 to −1·2)	4219 (3615 to 4970)	−9·9 (−14·6 to −5·4)	25 291 (16 673 to 42 462)	−31·3 (−50·7 to −4·5)
Ghana	4816 (3381 to 6556)	−25·7 (−40·2 to −4·5)	29 399 (25 428 to 35 109)	−11·4 (−15·8 to −6·9)	284 720 (204 913 to 399 893)	−32·2 (−48·1 to −7·6)
Guinea	3292 (2449 to 4599)	−33·5 (−50·5 to −10·8)	23 733 (20 357 to 28 377)	−20·7 (−26·0 to −15·4)	230 110 (165 486 to 326 940)	−46·2 (−63·1 to −18·0)
Guinea–Bissau	555 (382 to 882)	−33·2 (−49·2 to −4·3)	4194 (3608 to 4998)	−14·7 (−20·0 to −9·6)	37 215 (24 489 to 62 872)	−43·8 (−61·3 to −7·6)
Liberia	761 (529 to 1267)	−43·4 (−57·8 to −22·3)	4551 (3893 to 5492)	−24·5 (−28·8 to −20·0)	51 489 (34 353 to 94 219)	−57·0 (−71·4 to −32·8)
Mali	5116 (3277 to 8793)	−44·0 (−59·6 to −18·9)	40 794 (35 137 to 49 523)	−19·9 (−25·6 to −15·2)	379 397 (242 100 to 675 685)	−49·9 (−65·7 to −23·7)
Mauritania	511 (346 to 801)	−28·5 (−48·6 to 1·2)	4031 (3474 to 4788)	−7·9 (−13·0 to −2·4)	34 037 (23 359 to 54 867)	−23·7 (−46·2 to 16·1)
Niger	7872 (5274 to 11 933)	−54·1 (−71·3 to −22·7)	40 221 (34 064 to 47 868)	−24·9 (−31·0 to −18·5)	604 948 (398 660 to 951 399)	−63·2 (−78·3 to −29·1)
Nigeria	38 558 (24 792 to 65 133)	−35·6 (−56·8 to −4·1)	312 560 (258 893 to 390 273)	−19·1 (−25·2 to −12·4)	2 990 807 (1 923 124 to 5 086 003)	−39·6 (−62·0 to −1·8)
São Tomé and Príncipe	9 (5 to 18)	−41·0 (−59·6 to −12·6)	160 (137 to 191)	−4·3 (−9·6 to 1·8)	666 (396 to 1400)	−48·0 (−66·0 to −10·7)
Senegal	3210 (2194 to 5126)	−21·6 (−38·4 to 1·6)	28 069 (24 230 to 33 390)	−10·4 (−15·5 to −5·4)	214 387 (143 404 to 371 667)	−33·8 (−51·7 to −1·6)
Sierra Leone	1980 (1301 to 3546)	−35·2 (−51·5 to −12·1)	7602 (6467 to 9027)	−17·9 (−21·8 to −13·7)	143 172 (94 144 to 258 606)	−45·7 (−62·8 to −14·5)
Togo	1198 (901 to 1555)	−21·1 (−36·8 to −1·1)	10 582 (9050 to 12 668)	−9·7 (−15·5 to −3·5)	79 940 (60 188 to 106 973)	−30·3 (−48·0 to −7·4)
**Eastern sub-Saharan Africa**	**70 108 (57 232 to 87 791)**	**−36·7 (−46·2 to −21·6)**	**553 768 (484 889 to 645 363)**	**−13·9 (−16·3 to −11·6)**	**4 687 858 (3 852 788 to 5 933 910)**	**−45·1 (−55·7 to −25·4)**
Burundi	2489 (1851 to 3407)	−30·2 (−46·4 to −2·8)	16 158 (13 915 to 19 142)	−5·2 (−9·8 to −0·7)	174 529 (124 459 to 250 543)	−32·8 (−51·0 to 2·4)
Comoros	111 (83 to 157)	−30·3 (−46·3 to −4·3)	750 (654 to 882)	−16·6 (−20·7 to −12·5)	7070 (5172 to 10 294)	−36·8 (−54·1 to −7·8)
Djibouti	167 (116 to 253)	−27·4 (−46·6 to −1·9)	1190 (1018 to 1427)	−12·4 (−17·0 to −7·4)	10 950 (7158 to 17 089)	−39·0 (−58·5 to −7·4)
Eritrea	971 (715 to 1377)	−28·8 (−48·7 to −1·5)	6363 (5503 to 7508)	−12·6 (−17·3 to −8·4)	62 970 (44 821 to 93 279)	−31·7 (−50·8 to −0·1)
Ethiopia	17 313 (12 758 to 24 033)	−48·3 (−61·3 to −25·9)	192 617 (169 896 to 221 079)	−19·2 (−23·2 to −15·3)	1 061 705 (794 076 to 1 469 869)	−57·6 (−70·5 to −33·3)
Kenya	5483 (4462 to 7100)	−23·6 (−34·5 to −7·5)	48 931 (42 473 to 57 687)	−6·1 (−7·6 to −4·7)	350 334 (295 171 to 436 224)	−32·6 (−42·1 to −16·2)
Madagascar	4378 (3334 to 5912)	−20·1 (−37·0 to 2·1)	27 652 (23 781 to 32 824)	−12·9 (−17·9 to −8·1)	295 058 (219 216 to 397 920)	−26·8 (−45·3 to −0·9)
Malawi	3928 (2882 to 5469)	−43·3 (−60·8 to −17·4)	22 798 (19 514 to 27 114)	−26·1 (−30·7 to −22·2)	270 823 (195 011 to 394 647)	−55·0 (−71·7 to −24·8)
Mozambique	4633 (3410 to 6639)	−42·5 (−57·0 to −18·9)	33 880 (28 940 to 40 595)	−19·4 (−24·4 to −14·3)	326 488 (232 059 to 484 097)	−49·8 (−65·6 to −17·6)
Rwanda	1915 (1403 to 2741)	−42·8 (−59·3 to −10·5)	12 734 (10 900 to 15 234)	−24·2 (−29·0 to −19·6)	132 649 (95 579 to 195 871)	−48·3 (−64·7 to −11·5
Somalia	2621 (1986 to 3554)	−17·1 (−35·4 to 10·0)	11 283 (9 845 to 13 336)	−13·9 (−18·4 to −9·8)	170 445 (124 949 to 236 893)	−27·2 (−48·6 to 6·4)
South Sudan	4605 (3088 to 7248)	−17·9 (−39·6 to 15·9)	39 999 (34 383 to 47 455)	1·1 (−3·7 to 5·6)	334 897 (218 296 to 532 574)	−30·4 (−52·3 to 10·7)
Tanzania	8670 (6412 to 12 583)	−27·7 (−43·1 to −6·2)	62 761 (54 249 to 75 605)	−10·7 (−15·2 to −6·2)	578 159 (412 520 to 886 779)	−34·5 (−51·9 to −3·0)
Uganda	8755 (6408 to 12 434)	−35·0 (−50·7 to −11·1)	54 123 (46 589 to 65 194)	−15·4 (−20·6 to −10·7)	643 365 (460 045 to 947 999)	−41·4 (−60·5 to −7·4)
Zambia	4071 (2987 to 5515)	−35·9 (−54·7 to −14·3)	22 205 (19 055 to 26 291)	−15·5 (−19·6 to −10·7)	268 277 (195 922 to 371 965)	−50·9 (−67·2 to −25·0)
**Central sub-Saharan Africa**	**19 663 (14 550 to 28 206)**	**−22·4 (−42·2 to 8·3)**	**180 431 (155 424 to 216 821)**	**−5·9 (−9·9 to −1·8)**	**1 454 095 (1 037 645 to 2 179 955)**	**−29·6 (−49·6 to 11·0)**
Angola	3650 (2528 to 5450)	−34·2 (−56·7 to 7·3)	39 683 (33 971 to 47 745)	−6·8 (−11·2 to −1·8)	272 485 (185 885 to 419 387)	−40·1 (−61·0 to 6·7)
Central African Republic	1271 (868 to 1810)	−18·8 (−41·7 to 8·6)	8314 (7267 to 9838)	1·3 (−4·0 to 6·6)	90 246 (62 169 to 129 313)	−26·3 (−49·8 to 2·4)
Congo (Brazzaville)	570 (402 to 852)	−30·7 (−52·6 to 5·2)	6432 (5544 to 7613)	−3·6 (−8·4 to 1·3)	39 300 (27 061 to 60 832)	−33·5 (−53·3 to 2·0)
Democratic Republic of the Congo	13 920 (9478 to 21 499)	−16·9 (−40·3 to 23·5)	122 809 (105 870 to 147 459)	−6·1 (−11·2 to −0·8)	1 035 402 (665 043 to 1 689 370)	−25·1 (−51·7 to 30·6)
Equatorial Guinea	64 (41 to 100)	−65·6 (−80·2 to −39·9)	1022 (884 to 1203)	−12·7 (−17·7 to −7·5)	4276 (2934 to 6421)	−71·1 (−82·6 to −48·9)
Gabon	188 (133 to 298)	−36·6 (−57·4 to −3·7)	2171 (1884 to 2559)	−2·7 (−7·7 to 2·9)	12 385 (8693 to 20 650)	−42·8 (−61·4 to −10·1)

Data are n (95% UI) or percentage change in age-standardised rates (95%UI). DALYs= disability-adjusted life-years. SDI=Socio-demographic Index. UI=uncertainty interval.

Of the causes of incident meningitis, *Haemophilus influenzae* type b decreased the most (–49·1%), from 780 070 cases (95% UI 613 585–978 219) globally in 1990 to 397 297 cases (291 076–533 662) in 2016. Meningococcus was the leading cause of meningitis mortality in 1990 (192 833 deaths [95% UI 153 358–221 503] globally) but by 2016, the other causes category was the leading cause for both deaths (136 423 [112 682–178 022]) and incident cases (1·25 million [1·06–1·49]). Globally in 2016, 1·48 million (1·04–1·96) YLDs were due to meningitis compared with 21·87 million (18·20—28·28) DALYs, indicating that the contribution of mortality to meningitis burden is far greater than the contribution of disabling outcomes. Pneumococcus caused the largest number of YLDs (95% UI 634 458 [444 787–839 749]) in 2016, owing to its more severe long-term effects on survivors.

**Figure 2:**
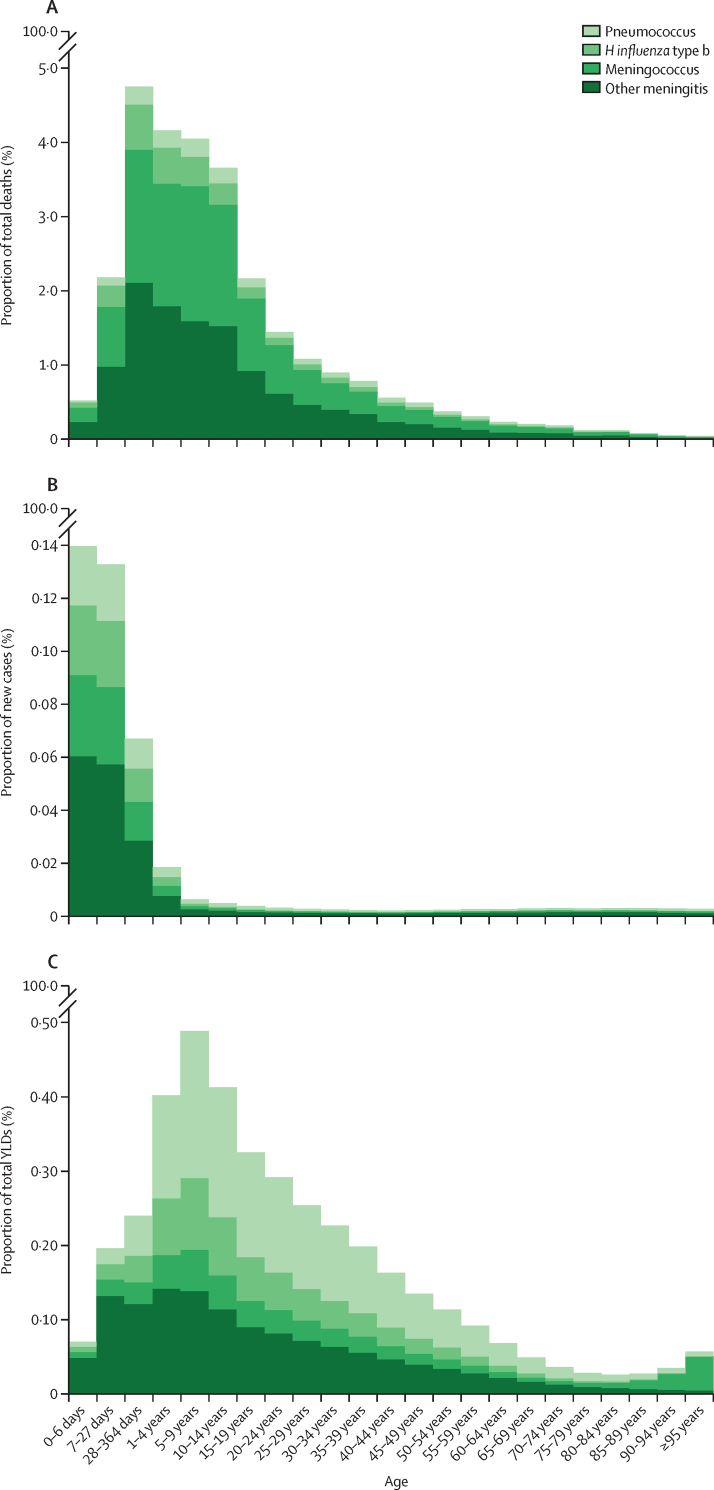
Cause fraction by age for deaths, incidence, and YLDs due to meningitis by cause globally and for both sexes, 2016 Cumulative cause fraction results (ie, proportion of total from all GBD causes) for deaths (A), incidence (B), and YLDs (C) for the four causes of meningitis considered by GBD: meningococcal due to *Neisseria meningitidis*, pneumococcal due to *Streptococcus pneumoniae, Haemophilus influenza* type b, and other meningitis (including other bacteria, viruses, and fungi). GBD=Global Burden of Diseases, Injuries, and Risk Factors Study. YLDs=years of life lived with disability.

**Figure 3:**
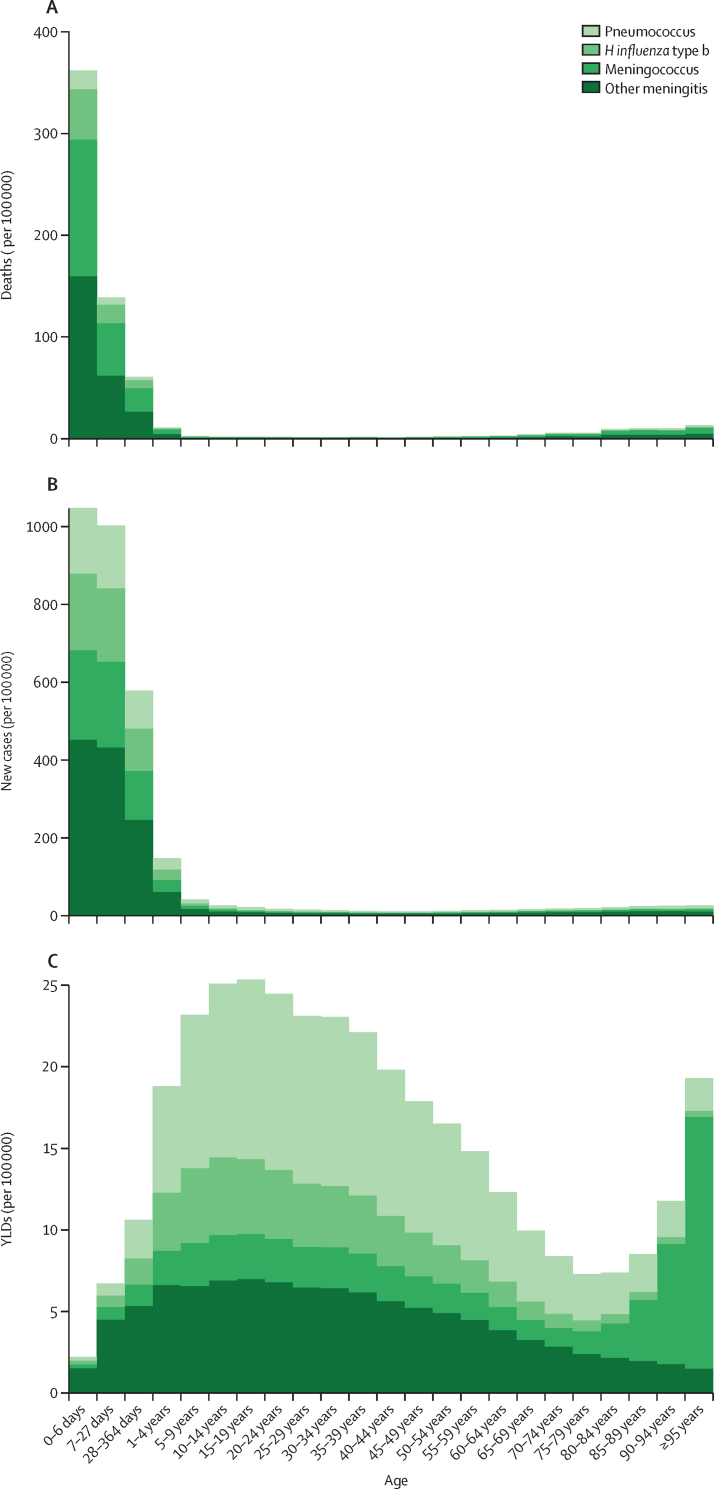
Age-specific rate of deaths, incidence, and YLDs due to meningitis by cause globally and for both sexes, 2016 Cumulative age-specific rates for cause-specific deaths (A), incidence (B), and YLDs (C) for the four causes of meningitis considered by GBD: meningococcal due to *Neisseria meningitidis*, pneumococcal due to *Streptococcus pneumoniae, Haemophilus influenza* type b, and other meningitis (including other bacteria, viruses, and fungi). GBD=Global Burden of Disease, Injuries, and Risk Factors Study. YLDs=years of life lived with disability.

**Figure 4:**
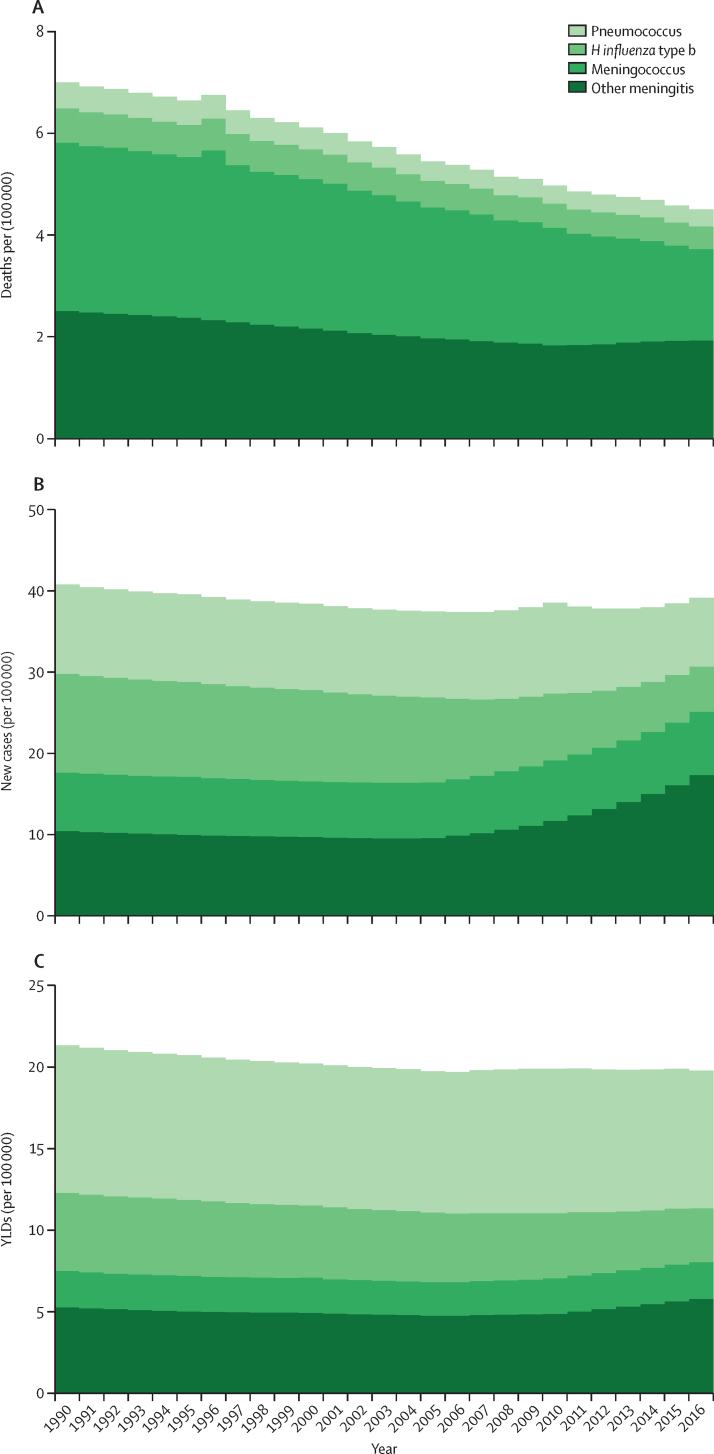
Age-standardised rates of deaths, incidence, and YLDs due to meningitis by cause globally and for both sexes, 1990–2016 Cumulative age-standardised rates from 1990 to 2016 for cause-specific deaths (A), incidence (B), and YLDs (C) for each of the four causes of meningitis considered by GBD: meningococcal due to *Neisseria meningitidis*, pneumococcal due to *Streptococcus pneumoniae, Haemophilus influenza* type b, and other meningitis (including other bacteria, viruses, and fungi). GBD=Global Burden of Disease, Injuries, and Risk Factors Study. YLDs=years of life lived with disability.

Trends in deaths, incidence, and YLDs for causes of meningitis showed distinct patterns with respect to proportion of burden by age (figure 2), rates by age (figure 3), and changes in age-standardised rates from 1990 to 2016 (figure 4). In younger ages, meningococcal meningitis and other meningitis were the predominant cause categories in incident cases and deaths. Death rate and incidence increased, as did YLD rates, in the oldest age groups, with other meningitis and meningococcal meningitis causing most of the burden in people 80 years and older. Global mortality decreased the most for meningococcal meningitis between 1990 and 2016 to the extent that, by 2016, the proportion of meningitis deaths in the other cause category (eg, due to *Listeria, Staphylococcus,* Gram-negative bacteria, fungal, and viral infections) was slightly higher in cause fraction to meningococcus. The time trend of incidence and YLDs was less stark. With decreasing incidence and YLD rates of *H influenzae* type b and pneumococcal meningitis, we estimated increasing rates of other meningitis, a trend that started around 2005. Although 54·0% (1·53 million [1·23–1·95]) of incident cases and 46·0% (146 322 [114 583–216 388]) of all deaths occurred in children younger than 5 years and risk of neurological disability was highest under 1 year of age, the accumulated disability burden was estimated to be highest in adolescents and young adults, with sequelae such as intellectual disability, epilepsy, vision impairment, and motor impairment.

**Figure 5:**
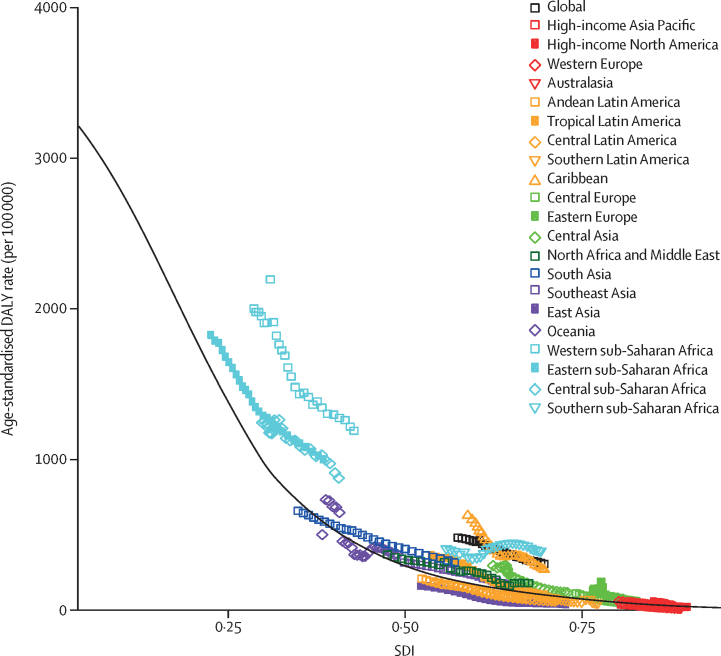
Age-standardised DALY rates for meningitis by 21 Global Burden of Disease regions by Socio-demographic Index, 1990–2016 The relationship between total disease burden due to all causes of meningitis, measured in DALYs, and sociodemographic development, as measured by the SDI. The average, or expected, rate of meningitis DALYs for a given level of SDI (black line) is calculated as the average meningitis DALY rate by age group, across all GBD estimation locations with that level of SDI. The observed values for each region for each year between 1990 and 2016 were aggregated from country results (coloured points). All points above the black line had higher meningitis DALY rates than expected based on SDI, while all those below the line had lower meningitis DALY rates than expected at that level of SDI. DALY=disability-adjusted life-years. GBD=Global Burden of Disease, Injuries, and Risk Factors Study. SDI=Socio-demographic Index.

Increasing SDI was consistently associated with lower age-standardised DALY rates due to meningitis (figure 5). Sub-Saharan Africa, without exception, had higher than expected age-standardised rates of meningitis DALYs, along with the Caribbean, north Africa, and the Middle East. Central Asia, southeast Asia, and eastern Europe all had rates in the 1990s that were higher than expected but improved compared with expectation by 2016. East Asia, high-income Asia Pacific, Andean Latin America, and central Latin America were the only regions with age-standardised meningitis DALY rates that were consistently lower than expected on the basis of SDI.

5·8% (4·5–7·0) of total meningitis DALYs were attributable to low birthweight and short gestation in 2016, with the highest rates of attributable DALYs occurring in the meningitis belt countries, Burundi, Malawi, Somalia, Zambia, and Afghanistan (appendix). All attributable burden was in neonates younger than 28 days, as described in the methods section.

## Discussion

Global mortality from meningitis has declined, but substantial disparities in geographical distribution and age groups persist. In 2016, nearly 0·6% of all-ages deaths and close to 3% of the total in children younger than 5 years were due to meningitis (figure 3A). The estimates indicate that, while most incident cases are now due to infections in the other meningitis category, meningococcal infections continue to be a major cause of meningitis mortality, and most long-term disability is due to the after-effects of pneumococcal infection. The peak age of incident meningitis is during the neonatal period, deaths are highest in children younger than 5 years, and meningitis death rates are highest in many of the same locations where overall younger than 5 years mortality is highest.^24^ Rates of disability among survivors are highest in the youngest children, with a peak of prevalent disability therefore accumulating in adolescents and young adults.

On the one hand, the overall figures provided by GBD 2016 are encouraging. Age-standardised death rates due to meningitis are estimated to have dropped globally by 21·0% from 1990 to 2016. On the other hand, the corresponding figures for other vaccine-responsive and preventable pathogen-based diseases have declined (eg, measles by 93·0%, tetanus by 90·7%, and syndrome-based diseases such as diarrhoea due to rotavirus by 57·9%),^18^ suggesting that progress in meningitis could be substantially faster. The need for accelerated action on meningitis across the globe was recognised in a high-level meeting in May, 2017, of more than 50 global experts, led by the Meningitis Research Foundation in collaboration with WHO, resulting in a commitment to formulate a global roadmap to defeat meningitis by 2030—a plan that required broad global commitments to strengthen systems for prevention, treatment, and surveillance.^25^

Vaccination is a successful intervention for preventing meningitis. *H influenzae* type b meningitis has historically been a common cause of childhood hearing loss and neurological disability, affecting 15%–30% of infected children, but widespread vaccination has virtually eliminated *H influenzae* type b in many high-income countries^26^ and has significantly reduced its incidence in many low-income countries.^27^ Widespread pneumococcal conjugate vaccination has also resulted in significant declines in invasive pneumococcal disease beyond the age groups targeted for vaccination, although post-licensure studies have detected an absence of efficacy against serotype 3.^28^ Pneumococcal conjugate vaccine generates herd protection by preventing nasopharyngeal carriage of bacteria targeted by the vaccine in people who are immunised, although corresponding increases in nasal carriage and infection by pneumococcal serotypes that are not covered has also been widely documented,^29^ suggesting that continued progress will require new and improved, higher-valency vaccines or vaccines that provide universal coverage against all pneumococcal strains.

Epidemics remain a persistent threat in the meningitis belt countries of sub-Saharan Africa. Six of the ten countries with the highest number of meningitis deaths are in the meningitis belt, where a high incidence of meningococcal meningitis in particular has been linked to climatic factors—low humidity, dry winds, and high levels of dust in the air.^30^ Mass vaccination for MenA with MenAfriVac across the belt led to steep declines in incidence of meningococcal meningitis, but epidemics due to MenC, MenW, MenX, and pneumococcus still occur. Nearly 18 000 cases of MenC were reported in Niger and Nigeria in 2017, the largest recorded epidemic.^17^ As affordable MenC polysaccharide conjugate vaccines used to control epidemics are being phased out of production^31^ and the potential remains high for epidemics from other serogroups, development of an affordable multivalent meningococcal vaccine is needed for mass vaccination campaigns and introduction into routine vaccination schedules.

Meningitis burden continues to be highest in resource-poor settings, which is not surprising given the confluence of factors predisposing to infections, epidemics, and poor outcomes. The proportion of survivors experiencing neurological disability is also estimated to be higher in people living in low-resource settings,^21^ owing to poorer access to acute syndromic care and fewer resources available for support of survivors with neurological sequelae. Even for people who can access services, out-of-pocket costs are the largest source of health expenditures in many low-income and middle-income countries; meningitis can lead to catastrophic health expenditures for poor households, which can lead to delays in seeking treatment or failure to seek treatment at all.^2^, ^4^, ^5^, ^11^, ^30^

In many areas of the world, especially rural areas, the capacity to do traditional diagnostic assays such as culture or PCR on CSF or blood samples is limited or non-existent. Limitations in capacity can be especially problematic in places where other infections, such as cerebral malaria,^32^ are common and can lead to delayed or missed diagnosis of bacterial or viral meningitis with resulting misclassification of deaths and under-representation of meningitis burden.^33^, ^34^ Additionally, diagnosis of meningitis in children, especially neonates, is difficult; the symptoms of meningitis in neonates are often subtle and mirror those seen in neonates with sepsis or other infections, resulting in potential under-reporting.^35^ Current assays also have important limitations: sensitivity to detect pathogens varies by pathogen; no assay is able to detect or characterise *N meningitidis* serogroup X; latex agglutination tests can be expensive and at times unreliable; and the widely used Pastorex agglutination test, although able to detect antigens from *H influenzae* type b, *S pneumoniae*, and *N meningitidis* serogroups A, C, Y, W, B, and group B *Streptococcus*, requires cold-chain storage.^36^ Country-specific regulations that allow only already overburdened medically-trained providers to do lumbar punctures should be reconsidered because such policies can delay needed care.^37^ Efforts should be made to enhance training for other providers to search comprehensively for the source of severe acute infections and do lumbar punctures as needed. To facilitate such a change in practice, development and deployment of an affordable, sensitive, and specific rapid diagnostic test for the main bacterial meningitis pathogens—one that does not require specialised laboratory equipment or refrigeration—is urgently needed.

Acceleration of declines in meningitis burden will require improved disease surveillance and more attention to the long-term implications and impairments for survivors. Enhanced national and regional surveillance would mean that countries can monitor meningitis locally and in turn improve the precision of global estimates. Although high-quality surveillance data are largely unavailable for most countries in sub-Saharan Africa,^38^ MenAfriNet is a counterpoint that can serve as a model for other countries. Comprising Mali, Niger, Burkina Faso, and Togo—four meningitis belt countries—MenAfriNet is a network that began doing high-quality case-based surveillance after MenA vaccine introduction.^39^ As MenAfriNet looks to expand, data sharing is crucial both to improve estimates and to show other countries how effective and accurate surveillance can be done. Improved awareness of the disease by populations and governments is also key, as is improved support for survivors and their families.^29^

Aside from GBD, no other global, cause-specific meningitis estimates are available across all age groups. WHO estimates cause-specific mortality due to meningitis using GBD cause-fractions for countries that do not have high-quality vital registration data and released cause-specific estimates for 2000 and 2016.^40^ WHO estimated only 141 deaths (95% CI 119–150) globally for meningitis in neonates younger than 28 days, 45 064 deaths (35 854–54 523) in infants aged 1–11 months, and 37 573 deaths (29 690–43 857) in children aged 1–4 years compared with GBD 2016, which estimated 8911 deaths (6074–12 348) in infants aged 0–6 days, 10 157 deaths (7812–15 141) in infants aged 7–27 days, 70 841 deaths (55 018–104 486) in infants aged 1–11 months, and 56 412 deaths (42 274–88 597) in children aged 1–4 years (see GBD Compare). The total number of all ages meningitis deaths estimated by WHO in 2016 (278 812 [226 461–323 217]), was also substantially lower than GBD 2016 (318 400 [95% UI 265 218–408 705]), although cause fractions were in closer agreement (0·49% for WHO in 2016; 0·58% [95% UI 0·48–0·75] for GBD 2016). Other publications reporting on meningitis epidemiology for particular pathogens or locations revealed some differences from the present analysis. First, in neonates, GBD 2016 estimated more meningococcus and less other meningitis than two studies that found most meningitis cases were due to group B *Streptococcus* and *Escherichia coli,* along with *Listeria* (in the high-income setting) or *S pneumonia* and *Klebsiella* (in the low-income and middle-income setting).^41^, ^42^ Second, combining mortality and non-fatal results to derive case-fatality ratios, GBD 2016 also estimated meningococcal meningitis to have a higher case-fatality rate than pneumococcal meningitis, a finding that contrasts with several studies that suggest lower case-fatality rate in meningococcal meningitis,^43^, ^44^ except occasionally in the setting of epidemics.^45^ These inconsistencies suggest that although case-fatality rate might be higher for meningococcal meningitis overall, case-fatality rate might vary by pathogen across specific age groups and geographic settings. It is important to note the comparatively high uncertainty of cause-specific meningitis estimates and to acknowledge the need to ensure all available evidence on mortality is used to inform non-fatal estimates and vice versa.

This study has several limitations. First, it shares the same limitations as the overall GBD 2016 study, which are detailed elsewhere.^23^, ^46^, ^47^, ^48^, ^49^ A major limitation is the relative paucity of data on distribution and severity of long-term sequelae in survivors, which for meningitis is especially notable given the high rates of neurological disability that can follow neonatal meningitis.^50^ Learning-related disabilities have not been included in GBD, potentially leading to burden underestimation in conditions such as meningitis. GBD criteria for risk-outcome pair criteria, which assess causal links from the perspective of risks rather than outcomes, are not ideal for assessing comparatively rare outcomes like meningitis. Second, cause-specific meningitis estimates have a high degree of uncertainty owing to a relative paucity of pathogen-specific data. Although surveillance data can be extremely variable in quality, comprehensiveness, and completeness, if methods could be developed to identify and incorporate high-quality surveillance data into GBD modelling, such data could further improve the temporal and geographical precision of overall and pathogen-specific estimates. Third, because GBD 2016 provides estimates for only three of the major bacterial causes of meningitis, important neonatal and infant pathogens, such as group B *Streptococcus, Listeria, Staphylococcus*, and *E coli*, are all grouped together; we aim to generate separate estimates for additional pathogens in future analyses. With release of a group B *Streptococcus* vaccine on the horizon, it will become crucial to obtain country-specific burden estimates to guide countries in planning and implementing vaccine strategies.^51^ Meningitis due to *Mycobacterium tuberculosis* and HIV-associated opportunistic infections were not included because GBD classifies them with the underlying diseases. Fourth, although SDI is a useful tool for exploring epidemiologic transition, it is incomplete because it does not capture important aspects of societal function, such as political stability, gender equity, urbanicity, technology penetrance, or infrastructure, which are important social determinants of health.

Meningitis burden remains unacceptably high in 2016, despite evidence that population-level vaccination campaigns can effectively prevent many causes of meningitis. Detailed country-specific meningitis burden estimates are crucial advocacy tools, empowering patients, caregivers, and civil society organisations to ask more from governments and funders to help target improved vaccination rates, to foster development of—and improve access to—rapid diagnostic tests, and to ensure that appropriate antibiotics for the most common causes of meningitis are included in the list of essential medicines. High rates of meningitis disability shine a spotlight on the need to strengthen health systems to provide additional educational support, aids and therapy for vision and hearing problems, and adaptation of the built environment for those with mobility issues. The inclusion of reliable surveillance data as inputs into GBD modelling will improve future meningitis estimates and will help prevent more people from dying from or having to live with the sequelae of meningitis.

Correspondence to: Dr Nicholas J Kassebaum, Global Health, Institute for Health Metrics and Evaluation, University of Washington, Seattle, WA, 98121, USA nickjk@uw.edu

or

Prof Joseph R Zunt, Departments of Neurology, Global Health, Medicine (Infectious Diseases) and Epidemiology, University of Washington, Seattle, WA 98104, USA jzunt@uw.edu

## References

[1] Müller O, Krawinkel M (2005). Malnutrition and health in developing countries. CMAJ.

[2] Baker M, McNicholas A, Garrett N (2000). Household crowding a major risk factor for epidemic meningococcal disease in Auckland children. Pediatr Infect Dis J.

[3] Miller L, Arakaki L, Ramautar A (2014). Elevated risk for invasive meningococcal disease among persons with HIV. Ann Intern Med.

[4] Hodgson A, Smith T, Gagneux S (2001). Risk factors for meningococcal meningitis in northern Ghana. Trans R Soc Trop Med Hyg.

[5] Battersby AJ, Knox-Macaulay HHM, Carrol ED (2010). Susceptibility to invasive bacterial infections in children with sickle cell disease. Pediatr Blood Cancer.

[6] Erdem H, Inan A, Guven E (2017). The burden and epidemiology of community-acquired central nervous system infections: a multinational study. Eur J Clin Microbiol Infect Dis.

[7] Kohli-Lynch M, Russell NJ, Seale AC (2017). Neurodevelopmental impairment in children after Group B streptococcal disease worldwide: systematic review and meta-analyses. Clin Infect Dis.

[8] Ramakrishnan M, Ulland AJ, Steinhardt LC, Moïsi JC, Were F, Levine OS (2009). Sequelae due to bacterial meningitis among African children: a systematic literature review. BMC Med.

[9] Hasbun R, Rosenthal N, Balada-Llasat JM (2017). Epidemiology of meningitis and encephalitis in the United States, 2011–2014. Clin Infect Dis.

[10] Hajjeh R, Mulholland K, Schuchat A, Santosham M (2013). Progress towards demonstrating the impact of Haemophilus influenzae type b conjugate vaccines globally. J Pediatr.

[11] Ayieko P, Akumu AO, Griffiths UK, English M (2009). The economic burden of inpatient paediatric care in Kenya: household and provider costs for treatment of pneumonia, malaria and meningitis. Cost Eff Resour Alloc.

[12] Ai J, Xie Z, Liu G (2017). Etiology and prognosis of acute viral encephalitis and meningitis in Chinese children: a multicentre prospective study. BMC Infect Dis.

[13] Conklin L, Loo JD, Kirk J (2014). Systematic review of the effect of pneumococcal conjugate vaccine dosing schedules on vaccine-type invasive pneumococcal disease among young children. Pediatr Infect Dis J.

[14] Trotter CL, Lingani C, Fernandez K (2017). Impact of MenAfriVac in nine countries of the African meningitis belt, 2010–15: an analysis of surveillance data. Lancet Infect Dis.

[15] International Vaccine Access Center (2018). VIEW-hub report: global vaccine introduction and implementation. http://www.view-hub.org/resourcesfile/VIEW-hubReports_Resources/2018_03/IVAC_VIEW-hub_Report%202018Mar.pdf.

[16] Cuevas LE, Jeanne I, Molesworth A (2007). Risk mapping and early warning systems for the control of meningitis in Africa. Vaccine.

[17] WHO (2018). Epidemic meningitis control in countries of the African meningitis belt, 2017. Wkly Epidemiol Rec.

[18] Naghavi M, Abajobir AA, Abbafati C (2017). Global, regional, and national age-sex specific mortality for 264 causes of death, 1980–2016: a systematic analysis for the Global Burden of Disease Study 2016. Lancet.

[19] Vos T, Abajobir AA, Abate KH (2017). Global, regional, and national incidence, prevalence, and years lived with disability for 328 diseases and injuries for 195 countries, 1990–2016: a systematic analysis for the Global Burden of Disease Study 2016. Lancet.

[20] Barber RM, Fullman N, Sorensen RJD (2017). Healthcare Access and Quality Index based on mortality from causes amenable to personal health care in 195 countries and territories, 1990–2015: a novel analysis from the Global Burden of Disease Study 2015. Lancet.

[21] Edmond K, Clark A, Korczak VS, Sanderson C, Griffiths UK, Rudan I (2010). Global and regional risk of disabling sequelae from bacterial meningitis: a systematic review and meta-analysis. Lancet Infect Dis.

[22] Salomon JA, Haagsma JA, Davis A (2015). Disability weights for the Global Burden of Disease 2013 study. Lancet Glob Health.

[23] GBD 2016 Risk Factors Collaborators (2017). Global, regional, and national comparative risk assessment of 84 behavioural, environmental and occupational, and metabolic risks or clusters of risks, 1990–2016: a systematic analysis for the Global Burden of Disease Study 2016. Lancet.

[24] Golding N, Burstein R, Longbottom J (2017). Mapping under-5 and neonatal mortality in Africa, 2000–15: a baseline analysis for the Sustainable Development Goals. Lancet.

[25] Meningitis Research Foundation (2017). A global vision for meningitis by 2030 and an action plan to get there. https://vaccineresources.org/details.php?i=2454.

[26] Wenger JD (1998). Epidemiology of Haemophilus influenzae type b disease and impact of Haemophilus influenzae type b conjugate vaccines in the United States and Canada. Pediatr Infect Dis J.

[27] WHO (2013). Haemophilus influenzae type b (Hib) vaccination position paper—September 2013. Wkly Epidemiol Rec.

[28] Andrews NJ, Waight PA, Burbidge P (2014). Serotype-specific effectiveness and correlates of protection for the 13-valent pneumococcal conjugate vaccine: a postlicensure indirect cohort study. Lancet Infect Dis.

[29] Weinberger DM, Malley R, Lipsitch M (2011). Serotype replacement in disease after pneumococcal vaccination. Lancet.

[30] Palmgren H (2009). Meningococcal disease and climate. Glob Health Action.

[31] Salisbury D, Ramsay M (2006). Chapter 22: Meningococcal. Immunisation against infectious disease.

[32] Gething PW, Casey DC, Weiss DJ (2016). Mapping *Plasmodium falciparum* mortality in Africa between 1990 and 2015. N Engl J Med.

[33] Misra UK, Kalita J, Prabhakar S, Chakravarty A, Kochar D, Nair PP (2011). Cerebral malaria and bacterial meningitis. Ann Indian Acad Neurol.

[34] Desmond NA, Nyirenda D, Dube Q (2013). Recognising and treatment seeking for acute bacterial meningitis in adults and children in resource-poor settings: a qualitative study. PLoS One.

[35] Pong A, Bradley JS (1999). Bacterial meningitis and the newborn infant. Infect Dis Clin North Am.

[36] Waite T, Telisinghe L, Gobin M (2014). Systematic review on rapid diagnostic tests for meningococcal meningitis disease in sub-Saharan Africa: WHO Protocol to inform the revision of meningitis outbreak response guidelines.

[37] Petti CA, Polage CR, Quinn TC, Ronald AR, Sande MA (2006). Laboratory medicine in Africa: a barrier to effective health care. Clin Infect Dis.

[38] Lingani C, Bergeron-Caron C, Stuart JM (2015). Meningococcal meningitis surveillance in the African Meningitis Belt, 2004–2013. Clin Infect Dis.

[39] Kambiré D, Soeters HM, Ouédraogo-Traoré R (2018). Early impact of 13-valent pneumococcal conjugate vaccine on pneumococcal meningitis-Burkina Faso, 2014–2015. J Infect.

[40] WHO (2000–2016). Disease burden and mortality estimates. Cause-specific mortality. http://www.who.int/healthinfo/global_burden_disease/estimates/en/.

[41] Furyk JS, Swann O, Molyneux E (2011). Systematic review: neonatal meningitis in the developing world. Trop Med Int Health.

[42] Okike IO, Johnson AP, Henderson KL (2014). Incidence, etiology, and outcome of bacterial meningitis in infants aged <90 days in the United Kingdom and Republic of Ireland: prospective, enhanced, national population-based surveillance. Clin Infect Dis.

[43] Davison K, Ramsay M (2003). The epidemiology of acute meningitis in children in England and Wales. Arch Dis Child.

[44] Boisier P, Maïnassara HB, Sidikou F, Djibo S, Kairo KK, Chanteau S (2007). Case-fatality ratio of bacterial meningitis in the African meningitis belt: we can do better. Vaccine.

[45] Brooks R, Woods CW, Benjamin DK, Rosenstein NE (2006). Increased case-fatality rate associated with outbreaks of Neisseria meningitidis infection, compared with sporadic meningococcal disease, in the United States, 1994–2002. Clin Infect Dis.

[46] GBD 2016 Causes of Death Collaborators (2017). Global, regional, and national age-sex specific mortality for 264 causes of death, 1980–2016: a systematic analysis for the Global Burden of Disease Study 2016. Lancet.

[47] GBD 2016 DALYs and HALE Collaborators (2017). Global, regional, and national disability-adjusted life-years (DALYs) for 333 diseases and injuries and healthy life expectancy (HALE) for 195 countries and territories, 1990–2016: a systematic analysis for the Global Burden of Disease Study 2016. Lancet.

[48] GBD 2016 Disease and Injury Incidence and Prevalence Collaborators (2017). Global, regional, and national incidence, prevalence, and years lived with disability for 328 diseases and injuries for 195 countries, 1990–2016: a systematic analysis for the Global Burden of Disease Study 2016. Lancet.

[49] GBD 2016 Mortality Collaborators (2017). Global, regional, and national under-5 mortality, adult mortality, age-specific mortality, and life expectancy, 1970–2016: a systematic analysis for the Global Burden of Disease Study 2016. Lancet.

[50] Anderson V, Anderson P, Grimwood K, Nolan T (2004). Cognitive and executive function 12 years after childhood bacterial meningitis: effect of acute neurologic complications and age of onset. J Pediatr Psychol.

[51] Madhi SA, Cutland CL, Jose L (2016). Safety and immunogenicity of an investigational maternal trivalent group B streptococcus vaccine in healthy women and their infants: a randomised phase 1b/2 trial. Lancet Infect Dis.

