# Correction to *Lancet Neurol* 2018; 17: 1061–82

**DOI:** 10.1016/S1474-4422(21)00381-1

**Published:** 2021-12

*GBD 2016 Meningitis Collaborators. Global, regional, and national burden of meningitis, 1990–2016: a systematic analysis for the Global Burden of Disease Study 2016.* Lancet Neurol *2018; **17:** 1061–82*—In this Article, the second sentence of the second paragraph in the Introduction section should read “The H influenzae type b vaccine is now part of immunisation programmes across 19 countries and the pneumococcal vaccine is part of immunisation programmes across 190 countries”. Additionally, Mohsen Naghavi should have been included in the GBD 2016 Meningitis Collaborators. The affiliations have also been updated. These corrections have been made to the online version as of Nov 17, 2021.

