# Supplementary material for: Global, regional, and national burden of meningitis, 1990–2016: a systematic analysis for the Global Burden of Disease Study 2016
Source: Lancet Neurol. 2018 Dec;17(12):1061–82. doi: 10.1016/S1474-4422(18)30387-9 (PMC6234314; doi:10.1016/S1474-4422(18)30387-9)
Supplement: Supplementary appendix [file mmc1.pdf]

THE LANCET
Neurology

Supplementary appendix

This appendix formed part of the original submission and has been peer reviewed. We post it as supplied by the authors.

Supplement to: GBD 2016 Meningitis Collaborators. Global, regional, and national burden of meningitis, 1990–2016: a systematic analysis for the Global Burden of Disease Study 2016. *Lancet Neurol* 2018; **17:** 1061–82.

Supplementary Methods and Results Appendix to:

Global, regional, and national burden of meningitis, incidence, mortality, and disability from 1990 to 2016: A systematic analysis for the GBD Global Burden of Disease 2016 study

Table of Contents

Summary of General Global Burden of Disease Study Methods 3

Appendix Table 1: GATHER checklist of information that should be included in reports of global health estimates, with description of compliance and location of information for GBD 2016. 6

Cause-specific mortality due to meningitis 7

Flowchart 7

Appendix Figure 1: Flowchart of meningitis mortality estimation 7

Input data 8

Appendix Table 2: International Classification of Disease codes used in estimating meningitis 8

Modelling strategy 10

Appendix Table 3: Covariates used for meningitis cause-of-death ensemble modelling (CODEm) ... 10

Appendix Figure 2: Map of meningitis belt countries (top; binary coding where 1= yes, 0 = no) and proportion of population living in meningitis belt (bottom), 2010 12

Appendix Table 4: Covariates and model fits for meningitis mortality proportion models 13

Central computation 13

Non-fatal health outcomes due to meningitis: incidence, prevalence, and years of life lived with disability (YLDs) 13

Flowchart and modeling overview 13

Appendix Figure 3: Flowchart of meningitis non-fatal burden estimation 14

Case definition 15

Input data 15

Appendix Table 5: Data availability for acute bacterial meningitis (overall) 16

Appendix Table 6: Aetiology-specific meningitis incidence proportion data availability 16

Modelling strategy 16

Appendix Table 6. Covariates and model fits for overall meningitis model 16

Appendix Table 8. Location-level covariates used for meningitis incidence etiology models 17

Severity splits 18

Disability weights and calculation of YLDs 19

Appendix Table 9. Health states, lay descriptions, and disability weights for long-term complications of meningitis 19

Supplementary Results 21

Appendix Figure 4: Global meningitis DALYs attributable to low birth weight and short gestation, all ages, both sexes, by year from 1990 to 2016 21

References 21

Summary of General Global Burden of Disease Study Methods

The Institute for Health Metrics and Evaluation with a growing collaboration of scientists produces annual updates of the Global Burden of Disease study. Estimates span the period from 1990 to the most recent completed year. By the time of the release of GBD 2016 in September 2017, there were over 2,700 collaborators in 132 countries who contributed to this global public good. GBD 2016 is compliant with the Guidelines for Accurate and Transparent Health Estimates Reporting (GATHER) statement[1] as illustrated in **Appendix Table 1**. All input data sources are available online on the Global Health Data Exchange (GHDx) using the GBD 2016 Input Data Sources Tool (http://ghdx.healthdata.org/gbd-2016/data-input-sources). Searches by "component" can be limited to "Causes of Death," "Nonfatal Health Outcomes," and "Risk Factors" and further specificity can be found by limiting to specific causes, which for the present analysis would include "Meningitis," "Pneumocococcal meningitis," "H influenza type B meningitis," "Meningococcal meningitis," or "Other meningitis." Searches can also be limited to specific geographies to return specific data source inputs.

Annual GBD updates allow incorporation of new data and method improvements to ensure that the most up-to-date information is available to policy makers in a timely fashion to help make resource allocation decisions. In this analysis, we have aggregated results from GBD 2016 for 15 disease and injury outcomes that are generally cared for by neurological services. These include infectious conditions (tetanus, meningitis, encephalitis), stroke, brain and other nervous system cancers, traumatic brain injury, and spinal cord lesion which are classified outside the more narrowly defined category of neurological disorders in GBD (ie, Alzheimer's disease and other dementias, Parkinson's disease, multiple sclerosis, motor neuron disease, idiopathic epilepsy, migraine, tension-type headache, and a rest category of less common other neurological disorders). Compared to a previous analysis based on GBD 2015,[2] we were able to add the non-fatal outcomes of traumatic brain injury and spinal cord lesion, and medication overuse headache is no longer included as a separate cause but quantified as a consequence of the underlying headache types.

In the methods section of this overview paper we present a summary of the general methods of the global burden of disease. In the accompanying disease-specific papers we concentrate on methods that are specific to each disorder. The guiding principle of GBD is to assess health loss due to mortality and disability comprehensively, where we define disability as any departure from full health. In GBD 2016, estimates were made for 195 countries and territories that are hierarchically organized into 21 regions and 7 super-regions, and 579 subnational locations, for 27 years starting from 1990, for 23 age groups and both sexes. Deaths were estimated for 264 diseases and injuries, while prevalence and incidence were estimated for 328 diseases and injuries. In order to allow meaningful comparisons between deaths and non-fatal disease outcomes as well as between diseases, the data on deaths and prevalence are summarised in a single indicator, the disability-adjusted life-year (DALY). DALYs are the sum of years of life lost (YLLs) and years lived with disability (YLDs). YLLs are estimated as the multiplication of counts of death and a standard, "ideal", remaining life expectancy at the age of death. The standard life expectancy is derived from the lowest observed mortality rates in any population in the world greater than 5 million.[3] YLDs are estimated as the product of prevalence of individual consequences of disease (or "sequelae") times a disability weight that quantifies the relative severity of a sequela as a number between zero (representing "full health") and 1 (representing death). Disability weights have been estimated in nine population surveys and an open-access internet survey in which respondents are asked to choose the

"healthier"[4] between random pairs of health states that are presented with a short description of the main features.

All-cause mortality rates are estimated from vital registration data in countries with complete coverage. For other countries, the probabilities of death before age 5 and between ages 15 and 60 are estimated from censuses and surveys asking mothers to provide a history of children ever born and those still alive, and surveys asking adults about siblings who are alive or have passed away. Using model life tables, these probabilities of death are transformed into age-specific death rates by location, year, and sex. GBD has collated a large database of cause of death data from vital registrations and verbal autopsy surveys in which relatives are asked a standard set of questions to ascertain the likely cause of death, supplemented with police and mortuary data for injury deaths in countries with no other data. For countries with vital registration data, the completeness is assessed with demographic methods based on comparing recorded deaths with population counts between two successive censuses. The cause of death information is provided in a large number of different classification systems based on versions of the International Classification of Diseases or bespoke classifications in some countries. All data are mapped into the disease and injury categories of GBD. All classification systems contain codes that are less informative because they lack a specific diagnosis (eg, unspecified cancer) or refer to codes that cannot be underlying cause of death (eg, low back pain or senility) or are intermediate causes (eg, heart failure or sepsis). Such deaths are redistributed to more precise underlying causes of death.[5] After these redistributions and corrections for under-registration, the data are analysed in CODEm (cause of death ensemble model), a highly systematised tool that runs many different models on the same data and chooses an ensemble of models that best reflects all the available input data. Models are chosen with variations in the statistical approach ("mixed effects" of spatiotemporal Gaussian Process Regression), in the unit of analysis (rates or cause fractions), and the choice of predictive covariates. The statistical performance of all models is tested by holding out 30% of the data and checking how well a model covers the data that were held out. To enforce consistency from CODEm, the sum of all cause-specific mortality rates is scaled to that of the all-cause mortality rates in each age, sex, location, and year category.

Non-fatal estimates are based on systematic reviews of published papers and unpublished documents, survey microdata, administrative records of health encounters, registries, and disease surveillance systems. Our Global Health Data Exchange (GHDx, http://ghdx.healthdata.org/) is the largest repository of health data globally. We first set a reference case definition and/or study method that best quantifies each disease or injury or consequence thereof. If there is evidence of a systematic bias in data that used different case definitions or methods compared to reference data we adjust those data points to reflect what its value would have been if measured as the reference. This is a necessary step if one wants to use all data pertaining to a particular quantity of interest rather than choosing a small subset of data of the highest quality only. DisMod-MR 2.1, a Bayesian meta-regression tool, is our main method of analyzing non-fatal data. It is designed as a geographical cascade where a first model is run on all the world's data, which produces an initial global fit and estimates coefficients for predictor variables and the adjustments for alternative study characteristics. The global fit adjusted by the values of random effects for each of seven GBD super-regions, the coefficients on sex and country predictors, are passed down as data to a model for each super-region together with the input data for that geography. The same steps are repeated going from super-region to 21 region fits and then to 195 fits by country and where applicable a further level down to subnational units. Below the global fit, all models are run separately by sex and for six time periods: 1990, 1995, 2000, 2005, 2010, and 2016. During each fit all data on prevalence,

incidence, remission (ie, cure rate) and mortality are forced to be internally consistent. For most diseases, the bulk of data on prevalence or incidence is at the disease level with fewer studies providing data on the proportions of cases of disease in each of the sequelae defined for the disease. The proportions in each sequela are pooled using DisMod-MR 2.1 or meta-analysis, or derived from analyses of patient-level datasets. The multiplication of prevalent cases for each disease sequela and the appropriate disability weight produces YLD estimates that do not yet take into account comorbidity. To correct for comorbidity, these data are used in a simulation to create hypothetical individuals in each age, sex, location, and year combination who experience no, one, or multiple sequelae simultaneously. We assume that disability weights are multiplicative rather than additive as this avoids assigning a combined disability weight value in any individual to exceed 1, ie, be worse than a "year lost due to death". This comorbidity adjustment leads to an average scaling down of disease-specific YLDs ranging from about 2% in young children up to 17% in oldest ages.

All our estimates of causes of death are categorical: each death is assigned to a single underlying cause. This has the attractive property that all estimates add to 100%. For risks, we use a different, "counterfactual" approach, ie, answering the question: "what would the burden have been if the population had been exposed to a theoretical minimum level of exposure to a risk". Thus, we need to define what level of exposure to a risk factor leads to the lowest amount of disease. We then analyse data on the prevalence of exposure to a risk and derive relative risks for any risk-outcome pair for which we find sufficient evidence of a causal relationship. Prevalence of exposure is estimated in DisMod-MR 2.1, using spatiotemporal Gaussian Process Regression, or from satellite imagery in the case of ambient air pollution. Relative risk data are pooled using meta-analysis of cohort, case-control and/or intervention studies. For each risk and outcome pair, we evaluate the evidence and judge if the evidence falls into the categories of "convincing" or "probable" as defined by the World Cancer Research Fund.[6] From the prevalence and relative risk results, population attributable fractions are estimated relative to the theoretical minimum risk exposure level (TMREL). When we aggregate estimates for clusters of risks, eg, metabolic or behavioural risks, we use a multiplicative function rather than simple addition and take into account how much of each risk is mediated through another risk. For instance, some of the risk of high body mass index is directly onto stroke as an outcome but much of its impact is mediated through high blood pressure, high cholesterol, or high fasting plasma glucose, and we would not want to double count the mediated effects when we estimate aggregates across risk factors.[7]

Uncertainty is propagated throughout all these calculations by creating 1,000 values for each prevalence, death, YLL, YLD, or DALY estimate and performing aggregations across causes and locations at the level of each of the 1,000 values for all intermediate steps in the calculation. The lower and upper bounds of the 95% uncertainty interval are the 25th and 975th values of the ordered 1,000 values. For all age-standardised rates, GBD uses a standard population calculated as the non-weighted average across all countries of the percentage of the population in each five-year age group for the years 2010 to 2035 from the United Nations Population Division's World Population Prospects (2012 revision).[8,9]

GBD uses a composite indicator or sociodemographic development, SDI, which reflects the geometric mean of normalised values of a location's income per capita, the average years of schooling in the population 15 and over, and the total fertility rate. Countries and territories are grouped into five quintiles of high, high-middle, middle, low-middle, and low SDI based on their 2016 values.[3]

Appendix Table 1: GATHER checklist of information that should be included in reports of global health estimates, with description of compliance and location of information for GBD 2016.

#	GATHER checklist item	Description of compliance	Reference
**Objectives and funding**			
1	Define the indicators, populations, and time periods for which estimates were made.	Narrative provided in paper and appendix describing indicators, definitions, and populations	Main text (Methods) and appendix
2	List the funding sources for the work.	Funding sources listed in paper	Summary (Funding)
**Data Inputs**			
For all data inputs from multiple sources that are synthesised as part of the study:			
3	Describe how the data were identified and how the data were accessed.	Narrative description of data seeking methods provided	Main text (Methods) and appendix
4	Specify the inclusion and exclusion criteria. Identify all ad-hoc exclusions.	Narrative about inclusion and exclusion criteria by data type provided; ad hoc exclusions in cause-specific write-ups	Main text (Methods) and appendix
5	Provide information on all included data sources and their main characteristics. For each data source used, report reference information or contact name/institution, population represented, data collection method, year(s) of data collection, sex and age range, diagnostic criteria or measurement method, and sample size, as relevant.	An interactive, online data source tool that provides metadata for data sources by component, geography, cause, risk, or impairment has been developed	Online data citation tools: http://ghdx.healthdata.org/gbd-2016
6	Identify and describe any categories of input data that have potentially important biases (e.g., based on characteristics listed in item 5).	Summary of known biases by cause included in appendix	Appendix
For data inputs that contribute to the analysis but were not synthesised as part of the study:			
7	Describe and give sources for any other data inputs.	Included in online data source tool	http://ghdx.healthdata.org/gbd-2016
For all data inputs:			
8	Provide all data inputs in a file format from which data can be efficiently extracted (e.g., a spreadsheet as opposed to a PDF), including all relevant meta-data listed in item 5. For any data inputs that cannot be shared due to ethical or legal reasons, such as third-party ownership, provide a contact name or the name of the institution that retains the right to the data.	Downloads of input data available through online data tools (visualization/ data query, Global Health Data Exchange [GHDx]); input data not in tools will be made available upon request	Online data visualisation tools, data query tools, and the Global Health Data Exchange
**Data analysis**			
9	Provide a conceptual overview of the data analysis method. A diagram may be helpful.	Flow diagrams of the overall methods and cause-specific modelling processes have been provided	Main text (Methods) and appendix
10	Provide a detailed description of all steps of the analysis, including mathematical formulae. This description should cover, as relevant, data cleaning, data pre-processing, data adjustments and weighting of data sources, and mathematical or statistical model(s).	Flow diagrams and methods write-ups for each cause, databases, and modelling processes have been provided	Main text (Methods) and appendix
11	Describe how candidate models were evaluated and how the final model(s) were selected.	Provided in methodological write-ups	Appendix
12	Provide the results of an evaluation of model performance, if done, as well as the results of any relevant sensitivity analysis.	Provided in methodological write-ups	Appendix
13	Describe methods for calculating uncertainty of the estimates. State which sources of uncertainty were, and were not, accounted for in the uncertainty analysis.	Appendix	Appendix
14	State how analytic or statistical source code used to generate estimates can be accessed.	Appendix	http://ghdx.healthdata.org/gbd-2016-code
**Results and Discussion**			
15	Provide published estimates in a file format from which data can be efficiently extracted.	GBD 2016 results available through online data tools, the Global Health Data Exchange (GHDx), and online data query tool	Main text, appendix, online data tools (visualization/ data query tools, GHDx)
16	Report a quantitative measure of the uncertainty of the estimates (e.g. uncertainty intervals).	Uncertainty provided with all results	Main text, appendix, online data tools (visualization/ data query tools, GHDx)

17	Interpret results in light of existing evidence. If updating a previous set of estimates, describe the reasons for changes in estimates.	Discussion of methodological changes between GBD rounds provided in the narrative of the manuscript and appendix	Main text (Methods and Discussion) and appendix
18	Discuss limitations of the estimates. Include a discussion of any modelling assumptions or data limitations that affect interpretation of the estimates.	Discussion of limitations provided in the narrative of the main paper, as well as in the methodological write-ups in the appendix	Main text (Limitations) and appendix

Cause-specific mortality due to meningitis

Descriptions of these methods were previously published as appendix material to the GBD 2016 manuscript, "Naghavi M, Abajobir AA, Abbafati C, et al. Global, regional, and national age-sex specific mortality for 264 causes of death, 1980–2016: a systematic analysis for the Global Burden of Disease Study 2016. *The Lancet* 2017; 390: 1151–210."

Flowchart

Appendix Figure 1 shows the flowchart of estimation for cause-specific mortality due to meningitis and four subtypes of meningitis: pneumococcal (due to *Streptococcus pneumoniae*), meningococcal (due to *Neisseria meningitides*), HiB (due to *Haemophilus influenzae* type B), and "other" (due to additional viral and bacterial agents). Overall methods across cause-specific mortality analyses are described in detail in the GBD 2016 cause of death manuscript.[10]

Appendix Figure 1: Flowchart of meningitis mortality estimation

This figure illustrates the step-by-step modeling process of meningitis mortality, including estimation of overall meningitis deaths and partitioning into each of the four subcause groups considered in GBD. Description of each of the main steps is in the following sections.

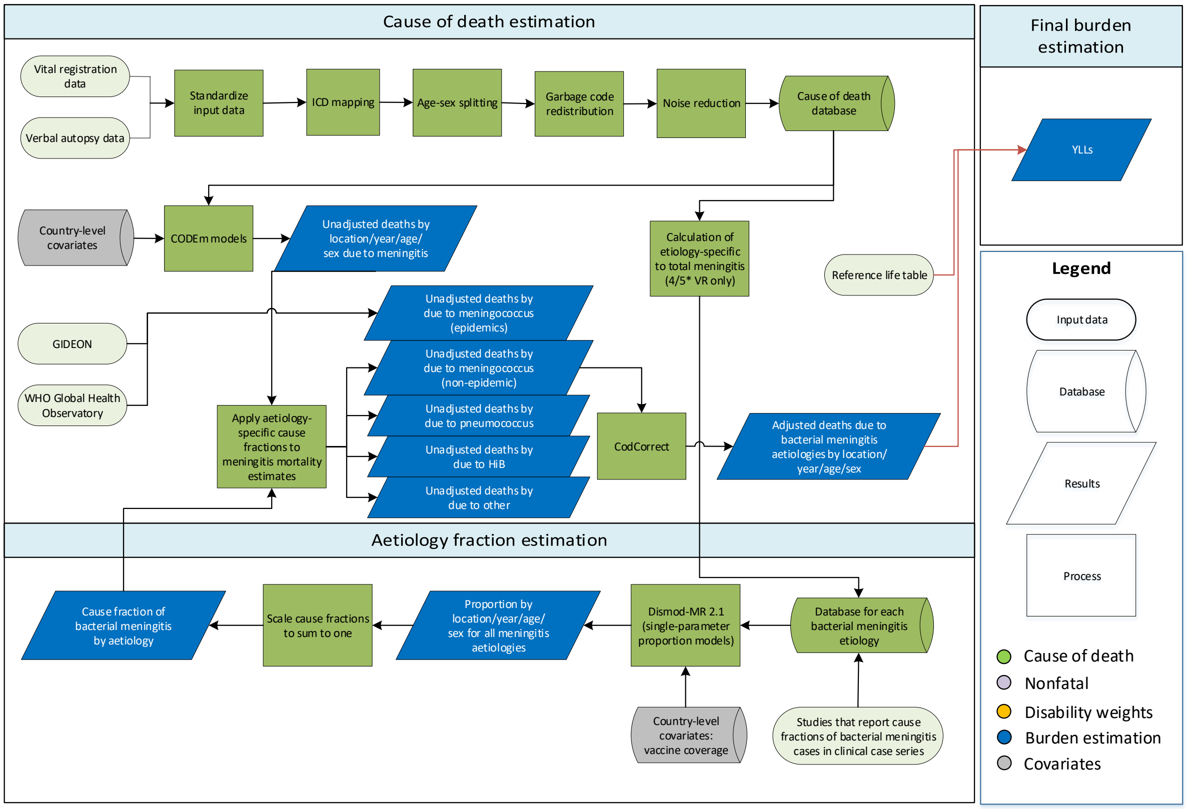

Input data

Input data for the overall meningitis model came from the cause of death (CoD) database, which includes vital registration (VR) and verbal autopsy (VA) data. ICD-9 and ICD-10 codes mapped to meningitis are shown in **Appendix Table 2**. In preparation for the CoD database, raw data were processed using a set of common algorithms, including 1) standardising data inputs, 2) ICD mapping, 3) age-sex splitting of data from combined age groups or both sexes, 4) correction of misclassification including redistribution of deaths coded to impossible, intermediate, and nonspecific causes to likely underlying diseases and injuries; and 5) implementation of Bayesian noise-reduction algorithms to simultaneously address stochastic variability in infrequent measurements and upward bias introduced by location-year-age-sex-specific instances where data show zero deaths (zeroes are dropped in log and logit models). We outliered data in instances where garbage code redistribution and noise reduction, in combination with small sample sizes, resulted in unreasonable cause fractions when compared to regional, super-regional, and global rates, and data that violated well-established time or age trends. Outliering methods were consistent across both VR and VA data.

Appendix Table 2: International Classification of Disease codes used in estimating meningitis

This table illustrates the International Classification of Diseases (ICD) codes where deaths were assigned to meningitis, by four subcauses considered, in GBD 2016. Any ICD codes not appearing in this list were not assigned to meningitis (e.g. tuberculous meningitis) except by redistribution from those codes were determined to be ill-defined (e.g. sepsis).

ICD-10	ICD-10 name	ICD-9	ICD-9 name
**Meningococcal meningitis**			
A39	Meningococcal infection	36	Meningococcal infection
A39.0	Meningococcal meningitis	36	Meningococcal meningitis
A39.1	Waterhouse-Friderichsen syndrome	36.1	Meningococcal encephalitis
A39.2	Acute meningococcemia	36.2	Meningococcemia
A39.3	Chronic meningococcemia	36.3	Waterhouse-Friderichsen syndrome, meningococcal
A39.4	Meningococcemia, unspecified	36.43	Meningococcal myocarditis
A39.5	Meningococcal heart disease	36.6	Meningococcal myocarditis
A39.50	Meningococcal carditis, unspecified	36.8	Other specified meningococcal infections
A39.51	Meningococcal endocarditis	36.81	Meningococcal optic neuritis
A39.52	Meningococcal myocarditis	36.82	Meningococcal arthropathy
A39.53	Meningococcal pericarditis	36.89	Other specified meningococcal infections
A39.8	Other meningococcal infections	36.9	Meningococcal infection unspecified
A39.81	Meningococcal encephalitis		
A39.82	Meningococcal retrobulbar neuritis		
A39.83	Meningococcal arthritis		
A39.84	Postmeningococcal arthritis		
A39.89	Other meningococcal infections		
A39.9	Meningococcal infection, unspecified		
**H. influenza type B meningitis**			
G00.0	Hemophilus meningitis	320	Hemophilus meningitis
**Pneumococcal meningitis**			
G00.1	Pneumococcal meningitis	320.1	Pneumococcal meningitis
**Other meningitis**			
A87	Viral meningitis	47	Meningitis due to enterovirus
A87.0	Enteroviral meningitis	47	Meningitis due to coxsackie virus
A87.1	Adenoviral meningitis	47.1	Meningitis due to echo virus
A87.2	Lymphocytic choriomeningitis	47.2	ECHO virus
A87.8	Other viral meningitis	47.3	ECHO virus
A87.9	Viral meningitis, unspecified	47.4	ECHO virus
D86.81	Sarcoid meningitis	47.8	Other specified viral meningitis
G00.2	Streptococcal meningitis	47.9	Unspecified viral meningitis
G00.3	Staphylococcal meningitis	48	Other enterovirus diseases of central nervous system
G00.8	Other bacterial meningitis	49	Other non-arthropod-borne viral diseases of central nervous system

G03	Meningitis due to other and unspecified causes	49	Lymphocytic choriomeningitis
G03.0	Nonpyogenic meningitis	49.1	Meningitis due to adenovirus
G03.1	Chronic meningitis	49.8	Other specified non-arthropod-borne viral diseases of central nervous system
G03.2	Benign recurrent meningitis [Mollaret]	49.9	Unspecified non-arthropod-borne viral diseases of central nervous system
G03.8	Meningitis due to other specified causes	320.2	Streptococcal meningitis
		320.3	Staphylococcal meningitis
		320.4	Tuberculous meningitis
		320.5	Meningococcal meningitis
		320.7	Meningitis in other bacterial diseases classified elsewhere
		320.8	Meningitis due to other specified bacteria
		320.81	Anaerobic meningitis
		320.82	Meningitis due to gram-negative bacteria, not elsewhere classified
		320.89	Meningitis due to other specified bacteria
		321	Cryptococcal meningitis
		321	Meningitis due to other organisms
		321.1	Meningitis in other fungal diseases
		321.2	Meningitis due to viruses not elsewhere classified
		321.3	Meningitis due to trypanosomiasis
		321.4	Meningitis in sarcoidosis
		321.5	Meningitis due to mumps virus
		321.6	Meningitis due to lymphocytic choriomeningitis virus
		321.7	Meningitis due to other and unspecified viruses
		321.8	Meningitis due to other nonbacterial organisms classified elsewhere
		322	Meningitis of unspecified cause
		322	Nonpyogenic meningitis
		322.1	Eosinophilic meningitis
		322.2	Chronic meningitis
		322.9	Meningitis, unspecified

Prior to GBD 2016, aetiologic attribution of meningitis deaths was informed by the proportion of incident cases due to each class of pathogen rather than the proportion of deaths (details in non-fatal estimation section). Viral meningitis mortality is rare except in the youngest age groups and is included with "other meningitis." Input data informing GBD 2016 aetiology-specific splits for meningitis came from two sources. First, we processed cause-specific VR mortality proportions (prepped as aetiology-specific deaths / total meningitis deaths) from the COD database, keeping only those from locations classified as data-rich according to the GBD 2016 data quality assessment.[10] Second, we completed a systematic literature review of meningitis mortality by cause for the first time in GBD 2016. The following search string was used in PubMed:

> ((("Meningitis"[MeSH] OR "Meningitis, pneumococcal"[MeSH] OR "Meningitis, Haemophilus"[MeSH] OR "Meningitis, Meningococcal"[MeSH] OR "Meningitis, viral"[MeSH] OR "Meningitis"[Title/Abstract]) AND (("etiology"[Title/Abstract] OR "causes" Title/Abstract] OR "cause pattern"[Title/Abstract] OR "aetiology"[Title/Abstract] OR "cause"[Title/Abstract]) AND ("fatality"[Title/Abstract] OR "mortality"[Title/Abstract] OR "death"[Title/Abstract]) AND 1985/01/01[PDAT]:3000/12/31[PDAT]) AND "humans"[MeSH])

The search was completed on November 21, 2016, yielding 1,290 hits for title and abstract review; 192 were selected for full text review, and 27 were extracted for use in aetiology proportion models. No limitation was placed on language of the studies. All proportion data were prepped as the number of aetiology-specific deaths divided by total meningitis deaths. The sample size was considered to be total

meningitis deaths. We outliered any data as implausible where the proportion of meningitis due to any of the four causes was less than 1% for all age groups and years.

Mortality due to epidemic outbreaks of meningococcal meningitis was quantified separately. This was added to cause-specific mortality estimates for the first time in GBD 2016 for two reasons. First, because epidemics of meningococcal meningitis have been reported to cause a non-trivial number of deaths, and second, because CODEm (described below) does not optimally capture the highly variable temporal patterns of epidemics. The Global Infectious Diseases and Epidemiology Network (GIDEON) served as the primary data source for collating meningococcal meningitis or meningococcal infection death reports.[11] For any year in which meningococcal meningitis deaths were recorded in a country or territory covered by the GBD, we directly extracted reported deaths from 1970 to 2016. When there were reporting gaps in cholera or meningococcal meningitis deaths over this period of time and the World Health Organization (WHO) annual cholera or meningitis reports had death reports for those years, we used the WHO reports.[12]

Modelling strategy

Overall meningitis was estimated using cause-of-death ensemble modelling (CODEm). Separate CODEm models were run for males and females, for under 5 years and 5-95+ years, and for all locations (ie, global) and for only the subset identified as data-rich (ie, 4- and 5-star COD data) for a total of eight CODEm models. All models used the same covariates and otherwise standard CODEm parameters. The final sex-specific models for deaths due to all bacterial meningitis were a hybridised model of separate global and data-rich models for each sex/age group combination. Location-level covariates used in CODEm models are shown in **Appendix Table 3** below and include 1) the proportion of population living in the meningitis belt, 2) proportion of children under 5 years who are underweight (<2 WAZ), 3) proportion of households with improved water, 4) the Healthcare Access and Quality (HAQ) Index,[13] 5) health system access, a composite of vaccine coverage and pregnancy services, 6) DTP3 vaccine coverage, 7) lag-distributed income (in international dollars per capita), 8) MenAfriVac vaccine coverage from 2010 to 2012, 9) improved sanitation proportion, and 10) maternal education (years per capita).

Appendix Table 3: Covariates used for meningitis cause-of-death ensemble modelling (CODEm)

Each of the following covariates were used in cause-of-death ensemble modeling of overall meningitis. Transform indicates what, if any, arithmetic transformation of the covariate was made prior to modeling. Direction indicates the required association between the covariate and the dependent variables of CODEm models (i.e. log-death rates or logit-cause fraction). Level refers to a hierarchical classification of covariates based on expected causal strength of association. Level 1 is strongly correlated causally, Level 2 may be ecologically associated or hypothesized, Level 3 are considered more distal determinants.

Covariate Name	Level	Direction	Transform
Proportion of population living in meningitis belt	1	+	
Underweight proportion (<5 yrs, <2 WAZ)	2	+	
Improved water (proportion of households)	2	-	Logit
Healthcare Access and Quality Index	2	-	
Health system access	2	-	
DTP3 coverage	3	-	
Lag distributed income ($I PC)	3	-	Log
MenAfriVac coverage	3	-	
Improved sanitation proportion	3	-	
Maternal education (Years PC)	3	-	

Evidence supporting covariate inclusion was varied. In a study of children admitted to 10 health centers in Latin America, children who were underweight were at increased risk of death from bacterial meningitis: compared to children of normal weight, those who were mildly underweight had a 1.98-fold increase in risk; the risk was 2.55-fold increased for moderately underweight, and 5.85-fold increased for children who were severely underweight.[14] In a study of patients aged 2 to 12 in a hospital in Angola, malnutrition was associated with higher risk of death from bacterial meningitis.[15] As illustrated in **Appendix Figure 2**, the "Meningitis Belt" was first defined as a "region south of the Sahara between latitudes 4 and 16°N" and was later expanded to include the Sahelian parts of Benin, Cameroon, Ethiopia, The Gambia, Ghana, Mali, and Senegal.[16] A study of the environmental characteristics of regions in Africa where meningitis occurs found that absolute humidity, dust, rainfall, land-cover type, and population densities were all associated with the location of epidemics.[17] A study of meningitis incidence in Michigan also found that population density was positively correlated with incidence of meningitis.[18] In a study of outbreaks of meningitis in Niger, as population density increased, there were higher incidence rates of meningitis.[19] Good sanitation is considered a preventive measure for contracting meningitis, especially for viral meningitis.[20] A study of Navajo Native Americans in the late 1960s and early 1970s discussed how meningitis in these communities could be due to limited access to water and poor sanitation.[21] A review of studies done on the epidemiology of bacterial meningitis in the United States and United Kingdom found that poverty, overcrowding, and low educational levels of parents are associated with increased incidence of meningitis.[22] A study of children admitted to a hospital in Melbourne, Australia, found that meningitis patients had lower IQ scores and academic abilities than patients without meningitis.[23] This finding is similar to other studies that found that higher levels of education are associated with better health outcomes in general.[24,25] In a meta-analysis of articles about the risks of specific sequelae caused by bacterial meningitis, the authors found that risk of disabling sequelae increased as gross national income decreased.[26] In a discussion of how socioeconomic variables relate to health, the author found that countries with lower GDP per capita have lower life expectancy than countries with higher GDP per capita.[27]

To obtain estimates for each of the four aetiologies of bacterial meningitis – meningococcal, pneumococcal, *H. influenzae* type B, and other bacterial – we ran separate proportion models in DisMod-MR 2.1 using VR proportion and systematic review data. Further details of the functionality of DisMod-MR 2.1 can be found in the supplemental appendix of the GBD 2016 manuscript on non-fatal health outcomes.[28] The meningococcal meningitis proportion model used two country-level covariates to inform the model – proportion of the population living within the meningitis belt, and proportion of the population covered by the meningococcal meningitis type A vaccine (an initiative called MenAfriVac). The pneumococcal meningitis model was informed by PCV3 vaccine coverage, and the H influenza type B meningitis model was informed by HiB3 vaccine coverage. The other meningitis proportion model was run with each of the three aetiology-specific vaccine coverage covariates, but with betas required to be negative. Summary statistics of model fits, covariates, beta values are shown in **Appendix Table 4** below and are available online at https://vizhub.healthdata.org/epi/. Because DisMod-MR 2.1 estimates in five-year intervals, the aetiological proportions for years between the intervals were interpolated at the draw level. The four proportion models were scaled to sum to 100% at the draw level for each location, age group, sex, and year combination. We applied these proportions to the total meningitis cause of death models to produce estimates for each of the four aetiologies.

Appendix Figure 2: Map of meningitis belt countries (top; binary coding where 1= yes, 0 = no) and proportion of population living in meningitis belt (bottom), 2010

The maps illustrate the peri-Sahelian countries of sub-Saharan Africa considered by GBD 2016 to be part of the meningitis belt. The top map is a binary (1 = yes, 0 = no) for if any part of the country is considered part of the meningitis belt. The bottom map shows which proportion of each country's population is estimated to reside within the meningitis belt. Countries in the meningitis belt include all of those countries south of the Sahara between latitudes 4 and 16^{0}N" as well as the Sahelian regions within Benin, Cameroon, Ethiopia, The Gambia, Ghana, Mali, and Senegal.

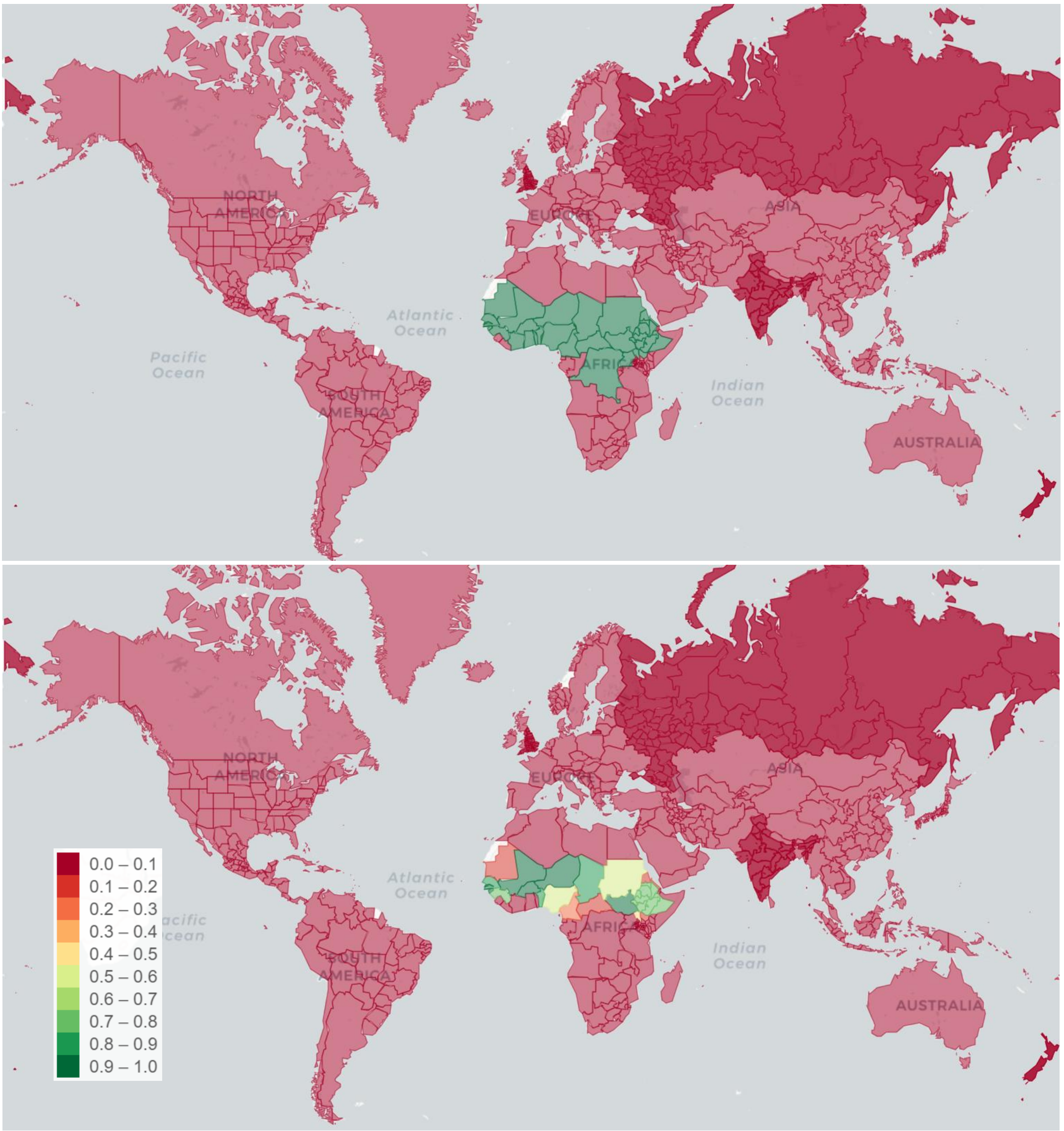

Appendix Table 4: Covariates and model fits for meningitis mortality proportion models

This table shows the study covariates and country covariates used in the four separate DisMod-MR 2.1 models used to estimate cause-specific meningitis mortality. All betas are calculated in log space and their exponentiated values are also shown. Each model's root mean squared error (RMSE) and in-sample data coverage are provided as measures of model performance.

Covariate	Type	Value	Exponentiated
Meningococcal meningitis mortality proportion			
Sex	Study-level (x-cov)	-0.025 (-0.039 — -0.01)	0.97 (0.96 — 0.99)
Meningitis belt (proportion)	Country-level	0.028 (0.00027 — 0.070)	1.03 (1.00 — 1.07)
MenAfriVac vaccine coverage (meningococcal type A vaccine)	Country-level	-1 (-2 — -0.0064)	0.37 (0.14 — 0.99)
	In-Sample RMSE	0.61499081	
	In-Sample Coverage	0.902020794	
H. influenzae type B meningitis mortality proportion			
Sex	Study-level (x-cov)	-0.0028 (-0.083 — 0.076)	1.00 (0.92 — 1.08)
Hib3 vaccine coverage (proportion)	Country-level	-0.017 (-0.067 — -0.00031)	0.98 (0.93 — 1.00)
	In-Sample RMSE	1.058748642	
	In-Sample Coverage	0.791602837	
Pneumococcal meningitis mortality proportion			
Sex	Study-level (x-cov)	0.0059 (-0.021 — 0.030)	1.01 (0.98 — 1.03)
PCV3 coverage (proportion)	Country-level	-0.00068 (-0.002 — -0.000059)	1.00 (1.00 — 1.00)
	In-Sample RMSE	0.91115243	
	In-Sample Coverage	0.896738549	
Other meningitis mortality proportion			
Sex	Study-level (x-cov)	0.013 (-0.0024 — 0.026)	1.01 (1.00 — 1.03)
Hib3 vaccine coverage (proportion)	Country-level	0.00055 (0.0000047 — 0.0012)	1.00 (1.00 — 1.00)
PCV3 coverage (proportion)	Country-level	0.0030 (0.00016 — 0.0091)	1.00 (1.00 — 1.01)
MenAfriVac vaccine coverage (meningococcal type A vaccine)	Country-level	1.01 (0.011 — 2.00)	2.76 (1.01 — 7.39)
	In-Sample RMSE	0.634024117	
	In-Sample Coverage	0.892378625	

Central computation

All aetiology-specific estimates, along with those of total non-epidemic and epidemic meningitis, were then processed through the GBD CoDCorrect algorithm that ensures the sum of all specific causes of death is equal to the all-cause mortality for each location, age group, sex, and year. The total number of age-specific deaths was then multiplied against the remaining global life expectancy at the age of death to calculate years of life lost (YLLs). The global life expectancy was calculated from a theoretical life table that aggregated the lowest observed death rates globally in each age group and was estimated to be 86.6 years at birth in GBD 2016. Further details of CoDCorrect and YLL calculation can be found in the supplemental appendix of the GBD 2016 manuscript on causes of death.[10]

Non-fatal health outcomes due to meningitis: incidence, prevalence, and years of life lived with disability (YLDs)

Descriptions of these methods were previously published as appendix material to the GBD 2016 manuscript, "Vos T, Abajobir AA, Abate KH, et al. Global, regional, and national incidence, prevalence, and years lived with disability for 328 diseases and injuries for 195 countries, 1990–2016: a systematic analysis for the Global Burden of Disease Study 2016. *The Lancet* 2017; 390: 1211–59."

Flowchart and modeling overview

Meningitis estimates were produced using a combination of DisMod-MR 2.1 models and post-modelling processes as illustrated in **Appendix Figure 3**. Overall meningitis was modelled in DisMod-MR 2.1 to

generate estimates of prevalence, incidence, and mortality that were internally consistent with each other as well as with those from the cause-specific mortality analysis above. Cause fractions for each of four aetiologies were also estimated using DisMod-MR 2.1 as single parameter proportion models. Viral meningitis was calculated using a ratio of bacterial to viral meningitis from hospital data. Meta-analyses of distribution and nature of long-term complications among survivors were used to generate incidence data for each type of long-term complication. These data were combined with other information on the mortality experience by type of long-term complication to generate age-specific estimates of long-term meningitis complications for each location, year, and sex and then proportionally split into different types of sequelae before calculating years of life lived with disability (YLDs).

Appendix Figure 3: Flowchart of meningitis non-fatal burden estimation

This flowchart illustrates all of the component datasets, estimation processes, intermediate results and central computation processes involved in estimation of cause-specific non-fatal disease burden due to meningitis.

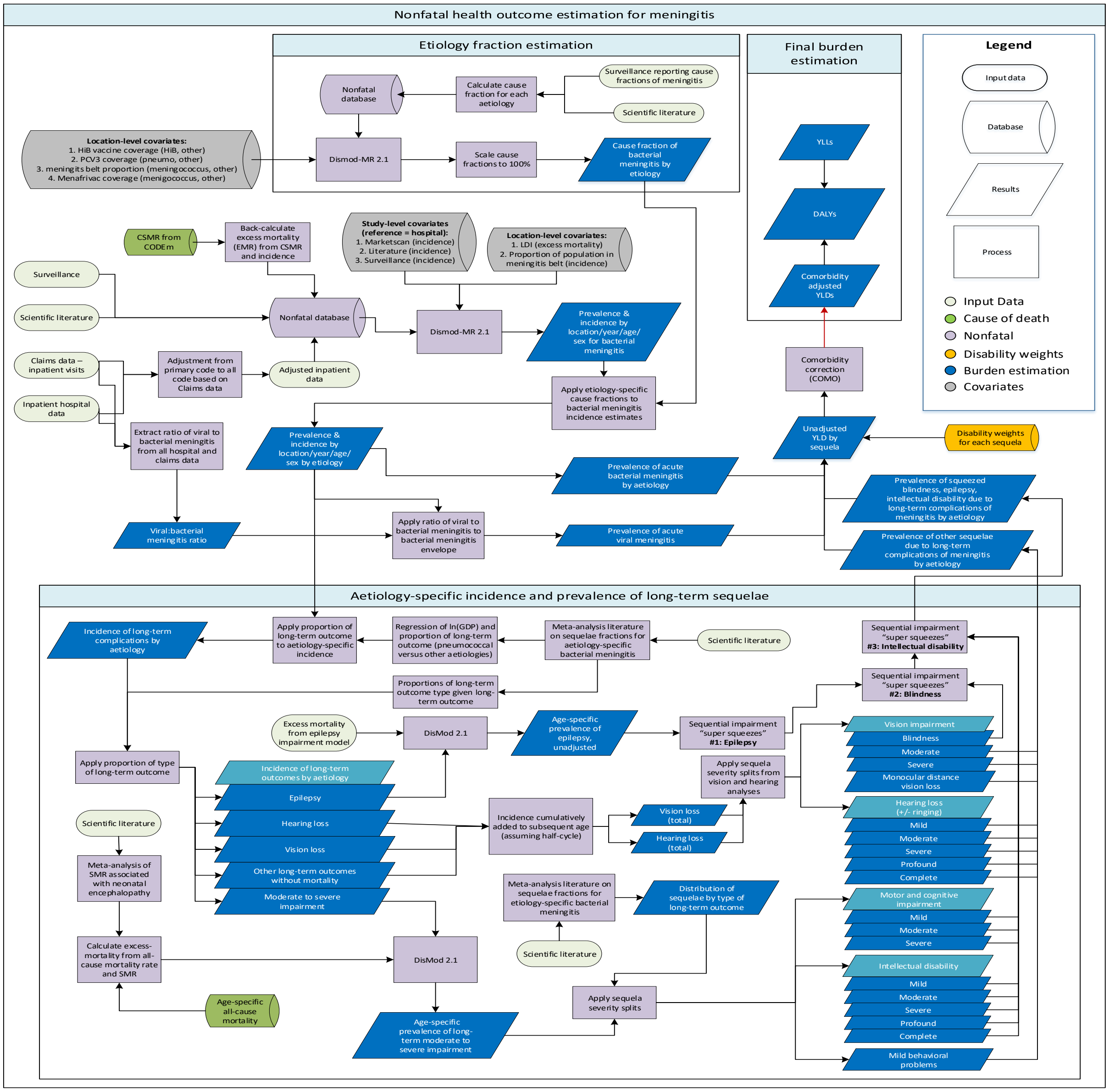

Case definition

Meningitis is a disease caused by inflammation of the meninges, the protective membrane surrounding the brain and spinal cord, and is typically caused by an infection in the cerebrospinal fluid. Symptoms include headache, fever, stiff neck, and sometimes seizures. Included in the GBD modelling were cases meeting ICD-9 and ICD-10 diagnostic criteria according to the mapping provided in **Appendix Table 1**.

Input data

In GBD 2010 and GBD 2013, systematic review of literature was conducted. The GBD 2010 search was conducted from 1980 to 2012 and the GBD 2013 search was extended to the end of 2013, with sufficient temporal overlap to ensure no missed studies and some revision of search strings to ensure no studies were missed. GBD 2010 searches were run in November 2011:

> Overall meningitis: ((meningitis[Title/Abstract] AND incidence[Title/Abstract]) AND ("0"[Date – Publication] : "2011"[Date – Publication])) **[2705 hits]**
>
> Etiology of incident cases: (((meningitis[Title/Abstract]) AND etiology[Title/Abstract]) AND "1990"[Publication Date] :"3000"[Publication Date]) **[676 hits]**

GBD 2013 search strings were run in December 2013:

> Overall meningitis: ((meningitis[Title/Abstract] AND incidence[Title/Abstract]) AND ("2011"[Date – Publication] : "2013"[Date – Publication])) **[221 hits]**
>
> Haemophilus influenza meningitis: (((HiB meningitis[Title/Abstract] OR haemophilus influenza[Title/Abstract]) AND incidence[Title/Abstract]) AND ("2010"[Date – Publication] : "2013"[Date – Publication])) **[22 hits]**
>
> Other meningitis: (((bacterial meningitis[Title/Abstract] OR viral meningitis[Title/Abstract]) AND incidence[Title/Abstract]) AND ("2010"[Date – Publication] : "2013"[Date – Publication])) **[66 hits]**
>
> Pneumococcal meningitis: (((pneumococcal meningitis[Title/Abstract] OR streptococcus pneumoniae[Title/Abstract]) AND incidence[Title/Abstract]) AND ("2010"[Date – Publication] : "2013"[Date – Publication])) **[214 hits]**
>
> Meningococcal meningitis: (((meningococcal meningitis[Title/Abstract] OR neisseria meningitis[Title/Abstract]) AND incidence[Title/Abstract]) AND ("2010"[Date – Publication] : "2013"[Date – Publication])) **[16 hits]**

Scientific literature studies informed five different components of the modelling process: 1) overall incidence of meningitis, 2) the proportion of meningitis due to each of the four bacterial aetiologies, 3) case fatality ratio and long-term mortality experience of those survivors with chronic complications of meningitis, 4) the proportion of survivors with long-term complications along with the nature and distribution of those long-term complications, and 5) the distribution of sequela severity by type of long-term outcome. Inclusion criteria stipulated that: (1) the publication year must be between 1980 and 2012; (2) "caseness" was based on diagnoses by antigen test, blood test, cerebrospinal fluid test, polymerase chain reaction test, or latex agglutination test; (3) sufficient information must be provided on study method and sample characteristics to assess the quality of the study; and (4) study samples must be representative of the general population. No limitation was set on the language of publication. The literature search was repeated for GBD 2015 only to capture case fatality ratio data, which were

converted to excess mortality assuming a duration of illness of 28 days. Surveillance data were used both to inform the overall incidence of meningitis and the aetiologic distribution of cases. ICD-coded hospital and claims data were used to inform overall incidence of meningitis as well as the distribution between bacterial and viral meningitis, as the latter is poorly captured in surveillance and literature sources. These were processed using standardised algorithms across all cause-specific analyses as described in the Supplemental Appendix of the GBD 2016 manuscript on non-fatal health outcomes.[28] **Appendix Table 5** shows the data availability for overall meningitis, and **Appendix Table 6** shows the data availability for aetiologies of meningitis.

The reference category for modelling was chosen to be hospital data as it was judged to be the most inclusive and representative across broad geographical areas. Most literature sources based on hospital data systems themselves included only those admissions where meningitis was the primary discharge diagnosis. We outliered any data that, even after crosswalking to the reference definition, were found to generate implausible geographic, temporal, or age patterns or were inconsistent with cause-specific mortality rate (CSMR) results from the above CoD analysis.

Appendix Table 5: Data availability for acute bacterial meningitis (overall)

Data dimension	Prevalence	Incidence	Mortality risk
Studies	0	70	70
Countries/subnationals	0	65/304	44/0
GBD world regions	0	20	17

Appendix Table 6: Aetiology-specific meningitis incidence proportion data availability

Data dimension	Pneumococcus (*S. pneumoniae*)	Meningococcus (*N. meningitides*)	*H. influenzae* type B	Other bacterial
Studies	67	62	68	60
Countries/subnationals	42/2	39/2	42/2	37/2
GBD world regions	18	17	18	16

Modelling strategy

Non-fatal outcomes were modelled using a combination of custom models and DisMod-MR 2.1, with only minor changes from the GBD 2015 modelling process. First, the overall incidence and prevalence of bacterial meningitis was modelled to estimate the short-term morbidity due to acute infection. Remission was bounded to equate to a duration (1/remission) of 4 weeks with a range ±2 weeks. Study-level covariates were used to crosswalk all non-reference data types (Literature, surveillance/ notification, Marketscan claims) to the reference definition of GBD 2016 inpatient-only hospital data. CSMR results from the cause-specific mortality analyses were incorporated into the model as data; excess mortality rate data were back-calculated as the quotient of CSMR and incidence using the remission prior above. Location-level covariates included the proportion of the population living in the meningitis belt (on incidence data) and log-transformed LDI (on excess mortality). Summary statistics of model fits, covariates, and beta values are shown in **Appendix Table 7** below and are available online at https://vizhub.healthdata.org/epi/.

Appendix Table 6. Covariates and model fits for overall meningitis model

This table shows the study covariates and country covariates used in the DisMod-MR 2.1 model used to estimate incidence and prevalence of all meningitis combined. All betas are calculated in log space and their exponentiated values are also shown. Each model's root mean squared error (RMSE) and in-sample data coverage are provided as measures of model performance.

Covariate effects				
Measure	Covariate	Type	Value	Exponentiated
Incidence	Sex	Study-level	0.19 (0.18 — 0.20)	1.21 (1.19 — 1.22)
Incidence	Literature	Study-level	-0.24 (-0.32 — -0.15)	0.79 (0.73 — 0.86)
Incidence	surveillance/notification data	Study-level	-1.23 (-1.58 — -0.89)	0.29 (0.21 — 0.41)
Incidence	inpatient-only Marketscan, year 2000	Study-level	-0.29 (-0.35 — -0.23)	0.75 (0.71 — 0.79)
Incidence	inpatient-only Marketscan, year 2010	Study-level	-0.32 (-0.37 — -0.27)	0.73 (0.69 — 0.76)
Incidence	inpatient-only Marketscan, year 2012	Study-level	-0.36 (-0.42 — -0.31)	0.69 (0.66 — 0.73)
Incidence	meningitis belt (proportion)	Country-level	1.52 (0.95 — 1.97)	4.56 (2.59 — 7.19)
Excess mortality rate	Sex	Study-level	0.057 (0.032 — 0.083)	1.06 (1.03 — 1.09)
Excess mortality rate	LDI (I$ per capita)	Country-level	-0.28 (-0.29 — -0.26)	0.76 (0.74 — 0.77)

Model fit statistics		
Measure	Type	Value
Incidence	In-Sample RMSE	1.782401701
Incidence	In-Sample Coverage (by integrand)	0.676206589
Excess mortality rate	In-Sample RMSE	0.772175376
Excess mortality rate	In-Sample Coverage (by integrand)	0.854969997
Cause-specific mortality rate	In-Sample RMSE	0.817405634
Cause-specific mortality rate	In-Sample Coverage (by integrand)	0.900718392

Incidence of bacterial meningitis were split into four aetiologies (pneumococcal, meningococcal, *H. influenzae* type B, and other bacterial meningitis) using four incidence proportion models run in DisMod-MR 2.1. Location-level covariates were similar to those used for meningitis mortality proportion models with two exceptions. MenAfriVac coverage was tested, but not used, for incidence proportion models owing to its producing implausible geographic trends outside of sub-Saharan Africa. Additionally, we did not identify any sex-specific input data on meningitis incidence proportions by aetiology, so all models calculated male-to-female ratios equal to 1. Results from these models were squeezed to sum to 1 at the draw level for each location, year, age, and sex. Summary model statistics are below. **Appendix Table 8** shows model performance and fit statistics.

Appendix Table 8. Location-level covariates used for meningitis incidence etiology models

This table shows the study covariates and country covariates used in the four separate DisMod-MR 2.1 models used to estimate cause-specific meningitis incidence. All betas are calculated in log space and their exponentiated values are also shown. Each model's root mean squared error (RMSE) and in-sample data coverage are provided as measures of model performance.

Covariate	Type	Value	Exponentiated
Meningococcal meningitis incidence proportion			
Sex	Study-level (x-cov)	0 (0 — 0)	1.00 (1.00 — 1.00)
Meningitis belt (proportion)	Country-level	0.57 (0.031 — 1.46)	1.76 (1.03 — 4.31)
	In-Sample RMSE	0.990	
	In-Sample Coverage	0.469	
H. influenzae type B meningitis incidence proportion			
Sex	Study-level (x-cov)	0 (0 — 0)	1.00 (1.00 — 1.00)
Hib3 vaccine coverage (proportion)	Country-level	-1.27 (-1.66 — -0.9)	0.28 (0.19 — 0.41)
	In-Sample RMSE	0.986	
	In-Sample Coverage	0.448	
Pneumococcal meningitis incidence proportion			
Sex	Study-level (x-cov)	0 (0 — 0)	1.00 (1.00 — 1.00)
PCV3 coverage (proportion)	Country-level	-0.85 (-1.92 — -0.033)	0.43 (0.15 — 0.97)
	In-Sample RMSE	0.643	
	In-Sample Coverage	0.570	
Other meningitis incidence proportion			
Sex	Study-level (x-cov)	0 (0 — 0)	1.00 (1.00 — 1.00)
Hib3 vaccine coverage (proportion)	Country-level	0.52 (0.046 — 0.99)	1.67 (1.05 — 2.68)

**PCV3 coverage (proportion)**	Country-level	1.06 (0.057 — 1.96)	2.90 (1.06 — 7.06)
**Meningitis belt (proportion)**	Country-level	-1.12 (-1.94 — -0.24)	0.33 (0.14 — 0.79)
	**In-Sample RMSE**	1.279	
	**In-Sample Coverage**	0.366	

Data for viral meningitis were only available from hospitals or USA claims data, and not from population studies, so incidence and prevalence of viral meningitis were extrapolated from bacterial meningitis incidence by applying age- and sex-specific ratios between bacterial and viral cases from a combination of hospital data and USA claims data. In addition to short-term sequelae as a result of acute bacterial and viral meningitis, we also modelled the long-term outcomes from bacterial meningitis infection.

Severity splits

We first split the long-term sequelae among survivors of acute infection. We calculated the acute-phase survivors by applying the excess mortality (calculated by the acute meningitis parent DisMod-MR 2.1 model) to the incidence of each aetiology. Excess mortality was converted to case fatality rate assuming a duration of 28 days using the equation

$$CFR = e^{-emr * \frac{1}{emr+rem}}$$

Where *emr* is excess mortality rate expressed as deaths per person-year of illness and *rem* is remission expressed as resolved cases per person-year of illness. The survivors were then subject for long-term sequelae by applying the post-discharge proportions of health consequences calculated by a meta-analysis by Edmond and colleagues.[26] We calculated the ratio of acute meningitis survivors that experience major long-term impairments for all aetiologies, and the ratio of minor impairments to major impairments for pneumococcal meningitis versus all other aetiologies because pneumococcal meningitis showed significantly higher risk of morbidity than other aetiologies. This ratio was based off a regression of log-transformed gross-domestic product (GDP) and ratio values from Edmond and colleagues. The regression is shown below:

$$y = -0.33590 \ln(GDP) + 1.15230$$

We used these two ratios to calculate the proportions of survivors who contract a long-term minor impairment and those who contract a long-term major impairment. The proportion with major impairments were further split using pooled proportions from Edmond and colleagues' analysis into specific major impairments, which were grouped into vision loss, hearing loss, moderate-to-severe cognitive impairments, and epilepsy.

The calculated incidence of long-term sequelae was then converted to prevalence by two different approaches. For the sequelae not associated with excess mortality, which were vision loss, hearing loss, intellectual disability, motor impairment, and behavioural problems, the incidence of each age was cumulatively added up to the subsequent age (assuming half-cycle) to construct prevalence at each age. If the sequela is associated with excess mortality (epilepsy and moderate-to-severe cognitive impairments), the calculated incidence was run through a second DisMod-MR 2.1 model, paired with two sources of excess mortality data: 1) from the epilepsy envelope DisMod-MR 2.1 model generated for GBD 2016, and 2) back-calculated from standardised mortality ratio data of long-term complications following neonatal encephalopathy from asphyxia and birth trauma, also as used in GBD 2016. This second set of DisMod-MR 2.1 models assumed zero remission. After calculation of sequela-specific prevalence, each of

vision loss, hearing loss, cognitive impairment, and epilepsy outcomes were scaled to match the totals for each condition as estimated across all causes in GBD 2016.

Disability weights and calculation of YLDs

The basis of the GBD disability weight survey assessments are lay descriptions of health states highlighting major functional consequences and symptoms. Disability weights (DW) were quantified via survey of more than 60,000 respondents in surveys conducted among the general population in nine countries (Bangladesh, Indonesia, Peru, Tanzania, USA, Hungary, Italy, Netherlands, and Sweden) and in an open web-based survey. Disability weights range between 0 (perfect health) and 1 (death). The DW for each health state was paired with a corresponding sequela-level prevalence result and multiplied to calculate years of life lived with disability (YLDs).[29] All acute meningitis cases were assigned a health state and corresponding DW for "infectious disease, acute episode, severe." Mild behavioural problems were approximated with the lay health state description for attention deficit hyperactivity disorder. Epilepsy DWs for two separate health states based on seizure frequency, $\geq$ 1 time per month and 1-11 times per year, were ensembled to generate a combined disability weight for epilepsy due to meningitis. The lay descriptions and disability weights for sequelae associated with long-term complications of meningitis are shown below in **Appendix Table 9**.

Appendix Table 9. Health states, lay descriptions, and disability weights for long-term complications of meningitis

This table shows all of the sequelae estimated as non-fatal outcomes of meningitis. As described above, the type, distribution, frequency, and natural history of survivors of meningitis is dependent on the cause of the meningitis.

Health state	Lay description: "This person... "	DW (95% CI)
Infectious disease, acute episode, severe	has a high fever and pain, and feels very weak, which causes great difficulty with daily activities.	0.133 (0.088-0.190)
Attention deficit hyperactivity disorder (Mild behaviour problems)	is hyperactive and has difficulty concentrating, remembering things, and completing tasks.	0.045 (0.028-0.066)
Hearing loss, mild	has great difficulty hearing and understanding another person talking in a noisy place (for example, on an urban street).	0.01 (0.004-0.019)
Hearing loss, mild, with ringing	has great difficulty hearing and understanding another person talking in a noisy place (for example, on an urban street), and sometimes has annoying ringing in the ears.	0.021 (0.012-0.036)
Hearing loss, moderate	is unable to hear and understand another person talking in a noisy place (for example, on an urban street), and has difficulty hearing another person talking even in a quiet place or on the phone.	0.027 (0.015-0.042)
Hearing loss, moderate, with ringing	is unable to hear and understand another person talking in a noisy place (for example, on an urban street), has difficulty hearing another person talking even in a quiet place or on the phone, and has annoying ringing in the ears for 5 minutes at a time, almost every day.	0.074 (0.049-0.107)
Hearing loss, severe	is unable to hear and understand another person talking, even in a quiet place, and unable to take part in a phone conversation. Difficulties with communicating and relating to others cause emotional impact at times (for example worry or depression).	0.158 (0.105-0.227)
Hearing loss, severe, with ringing	is unable to hear and understand another person talking, even in a quiet place, is unable to take part in a phone conversation, and has annoying ringing in the ears for more than 5 minutes at a time, almost every day. Difficulties with communicating and relating to others cause emotional impact at times (for example worry or depression).	0.261 (0.175-0.36)
Hearing loss, profound	is unable to hear and understand another person talking, even in a quiet place, is unable to take part in a phone conversation, and has great difficulty hearing anything in any other situation. Difficulties with communicating and relating to others often cause worry, depression or loneliness.	0.204 (0.134-0.288)
Hearing loss, profound, with ringing	is unable to hear and understand another person talking, even in a quiet place, is unable to take part in a phone conversation, has great difficulty hearing anything in any other situation, and has annoying ringing in the ears for more than 5 minutes at a time, several times a day. Difficulties with communicating and relating to others often cause worry, depression, or loneliness.	0.277 (0.182-0.387)

Hearing loss, complete	cannot hear at all in any situation, including even the loudest sounds, and cannot communicate verbally or use a phone. Difficulties with communicating and relating to others often cause worry, depression or loneliness.	0.215 (0.144-0.307)
Hearing loss, complete, with ringing	cannot hear at all in any situation, including even the loudest sounds, and cannot communicate verbally or use a phone, and has very annoying ringing in the ears for more than half of the day. Difficulties with communicating and relating to others often cause worry, depression or loneliness.	0.316 (0.212-0.435)
Intellectual disability, borderline	is slow in learning at school. As an adult, the person has some difficulty doing complex or unfamiliar tasks but otherwise functions independently.	0.011 (0.005-0.02)
Intellectual disability, mild	has low intelligence and is slow in learning at school. As an adult, the person can live independently, but often needs help to raise children and can only work at simple supervised jobs.	0.043 (0.026-0.064)
Motor impairment, mild	has some difficulty in moving around but is able to walk without help.	0.01 (0.005-0.019)
Motor impairment, moderate	has some difficulty in moving around, and difficulty in lifting and holding objects, dressing and sitting upright, but is able to walk without help.	0.061 (0.04-0.089)
Motor impairment, severe	is unable to move around without help, and is not able to lift or hold objects, get dressed or sit upright.	0.402 (0.268-0.545)
Motor plus cognitive impairments, mild	has some difficulty in moving around but is able to walk without help. The person is slow in learning at school. As an adult, the person has some difficulty doing complex or unfamiliar tasks but otherwise functions independently.	0.031 (0.018-0.05)
Motor plus cognitive impairments, moderate	has some difficulty in moving around, holding objects, dressing and sitting upright, but can walk without help. The person has low intelligence and is slow in learning to speak and to do simple tasks. As an adult, the person requires support to live independently and raise children and can only work at simple supervised jobs.	0.203 (0.134-0.29)
Motor plus cognitive impairments, severe	cannot move around without help, and cannot lift or hold objects, get dressed or sit upright. The person also has very low intelligence, speaks few words, and needs constant supervision and help with all daily activities.	0.542 (0.374-0.702)
Epilepsy	>= 1 time per month: has sudden seizures one or more times each month, with violent muscle contractions and stiffness, loss of consciousness, and loss of urine or bowel control. Between seizures the person has memory loss and difficulty concentrating. 1-11 times per year: has sudden seizures two to five times a year, with violent muscle contractions and stiffness, loss of consciousness, and loss of urine or bowel control.	>= 1 per month: 0.552 (0.375-0.71) 1-11 per year: 0.263 (0.173-0.367)
Distance vision blindness	is completely blind, which causes great difficulty in some daily activities, worry and anxiety, and great difficulty going outside the home without assistance.	0.187 (0.124-0.26)
Distance vision blindness, monocular	is blind in one eye and has difficulty judging distances.	0.017 (0.009-0.029)

Supplementary Results

Appendix Figure 4: Global meningitis DALYs attributable to low birth weight and short gestation, all ages, both sexes, by year from 1990 to 2016

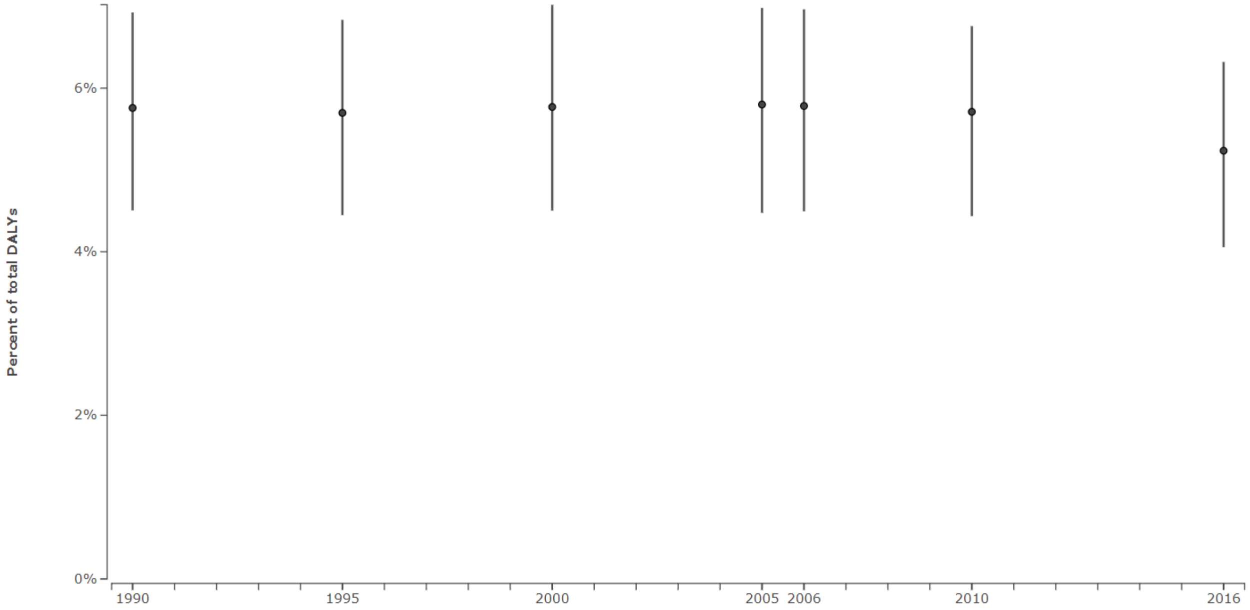

References

1 Stevens GA, Alkema L, Black RE, *et al.* Guidelines for Accurate and Transparent Health Estimates Reporting: the GATHER statement. *Lancet* 2016; published online June 28.

2 GBD 2015 Neurological Disorders Collaborator Group. Global, regional, and national burden of neurological disorders during 1990-2015: a systematic analysis for the Global Burden of Disease Study 2015. *Lancet Neurol* 2017; **16**: 877–97.

3 GBD 2016 Mortality Collaborators. Global, regional, and national under-5 mortality, adult mortality, age-specific mortality, and life expectancy, 1970-2016: a systematic analysis for the Global Burden of Disease Study 2016. *Lancet* 2017; **390**: 1084–150.

4 Salomon JA, Haagsma JA, Davis A, *et al.* Disability weights for the Global Burden of Disease 2013 study. *Lancet Glob Health* 2015; **3**: e712-723.

5 GBD 2016 Causes of Death Collaborators. Global, regional, and national age-sex specific mortality for 264 causes of death, 1980-2016: a systematic analysis for the Global Burden of Disease Study 2016. *Lancet* 2017; **390**: 1151–210.

6 American Institute for Cancer Research. Food, nutrition, physical activity, and the prevention of cancer: a global perspective. Washington, DC: American Institute for Cancer Research, 2007.

7 GBD 2016 Risk Factors Collaborators. Global, regional, and national comparative risk assessment of 84 behavioural, environmental and occupational, and metabolic risks or clusters of risks, 1990-2016: a systematic analysis for the Global Burden of Disease Study 2016. *Lancet* 2017; **390**: 1345–422.

8 GBD 2013 Mortality and Causes of Death Collaborators. Global, regional, and national age-sex specific all-cause and cause-specific mortality for 240 causes of death, 1990-2013: a systematic analysis for the Global Burden of Disease Study 2013. *Lancet* 2015; **385**: 117–71.

9 United Nations Department of Economics and Social Affairs Population Division. World Population Prospects: The 2012 Revision. http://esa.un.org/unpd/wpp/Documentation/publications.htm (accessed Nov 4, 2014).

10 Naghavi M, Abajobir AA, Abbafati C, *et al.* Global, regional, and national age-sex specific mortality for 264 causes of death, 1980–2016: a systematic analysis for the Global Burden of Disease Study 2016. *The Lancet* 2017; **390**: 1151–210.

11 Bacterial Meningitis: Global Status. GIDEON - Global Infectious Diseases and Epidemiology Online Network. https://www.gideononline.com/ebooks/disease/bacterial-meningitis-global-status/ (accessed April 6, 2018).

12 GHO | By category | Meningococcal meningitis. WHO. http://apps.who.int/gho/data/node.main.178?lang=en (accessed April 6, 2018).

13 Barber RM, Fullman N, Sorensen RJD, *et al.* Healthcare Access and Quality Index based on mortality from causes amenable to personal health care in 195 countries and territories, 1990–2015: a novel analysis from the Global Burden of Disease Study 2015. *The Lancet* 2017; **390**: 231–66.

14 Roine I, Weisstaub G, Peltola H. Influence of Malnutrition on the Course of Childhood Bacterial Meningitis. *The Pediatric Infectious Disease Journal* 2010; **29**: 122–5.

15 Pelkonen T, Roine I, Monteiro L, *et al.* Risk Factors for Death and Severe Neurological Sequelae in Childhood Bacterial Meningitis in Sub-Saharan Africa. *Clinical Infectious Diseases* 2009; **48**: 1107–10.

16 Cuevas LE, Jeanne I, Molesworth A, *et al.* Risk mapping and early warning systems for the control of meningitis in Africa. *Vaccine* 2007; **25**: A12–7.

17 Molesworth AM, Cuevas LE, Connor SJ, Morse AP, Thomson MC. Environmental Risk and Meningitis Epidemics in Africa. *Emerg Infect Dis* 2003; **9**: 1287–93.

18 Greene SK, Schmidt MA, Stobierski MG, Wilson ML. Spatio-temporal pattern of viral meningitis in Michigan, 1993-2001. *Journal of Geographical Systems* 2005; **7**: 85–99.

19 Djibo S, Nicolas P, Alonso J -M, *et al.* Outbreaks of serogroup X meningococcal meningitis in Niger 1995–2000. *Tropical Medicine & International Health* 2003; **8**: 1118–23.

20 Davison K, Ramsay M. The epidemiology of acute meningitis in children in England and Wales. *Arch Dis Child* 2003; **88**: 662–4.

21 Coulehan JL, Michaels RH, Williams KE, *et al.* Bacterial meningitis in Navojo Indians. *Public Health Rep* 1976; **91**: 464–8.

22 Ronald G. EPIDEMIOLOGY OF BACTERIAL MENINGITIS. *Infectious Disease Clinics of North America* 1999; **13**: 515–25.

23 Grimwood K, Anderson P, Anderson V, Tan L, Nolan T. Twelve year outcomes following bacterial meningitis: further evidence for persisting effects. *Archives of Disease in Childhood* 2000; **83**: 111–6.

24 Kimbro RT, Bzostek S, Goldman N, Rodríguez G. Race, Ethnicity, And The Education Gradient In Health. *Health Affairs* 2008; **27**: 361–72.

25 Marmot M. Social determinants of health inequalities. *The Lancet* 19; **365**: 1099–104.

26 Edmond K, Clark A, Korczak VS, Sanderson C, Griffiths UK, Rudan I. Global and regional risk of disabling sequelae from bacterial meningitis: a systematic review and meta-analysis. *Lancet Infect Dis* 2010; **10**: 317–28.

27 Fuchs VR. Reflections on the socio-economic correlates of health. *Journal of health economics*; **23**: 653–61.

28 Vos T, Abajobir AA, Abate KH, *et al.* Global, regional, and national incidence, prevalence, and years lived with disability for 328 diseases and injuries for 195 countries, 1990–2016: a systematic analysis for the Global Burden of Disease Study 2016. *The Lancet* 2017; **390**: 1211–59.

29 Salomon JA, Haagsma JA, Davis A, *et al.* Disability weights for the Global Burden of Disease 2013 study. *The Lancet Global Health* 2015; **3**: e712–23.
